# Proteomic Analysis of Tardigrades: Towards a Better Understanding of Molecular Mechanisms by Anhydrobiotic Organisms

**DOI:** 10.1371/journal.pone.0009502

**Published:** 2010-03-03

**Authors:** Elham Schokraie, Agnes Hotz-Wagenblatt, Uwe Warnken, Brahim Mali, Marcus Frohme, Frank Förster, Thomas Dandekar, Steffen Hengherr, Ralph O. Schill, Martina Schnölzer

**Affiliations:** 1 Functional Proteome Analysis, German Cancer Research Center, Heidelberg, Germany; 2 Department of Molecular Biology and Functional Genomics, University of Applied Sciences Wildau, Wildau, Germany; 3 Department of Bioinformatics, University of Würzburg, Würzburg, Germany; 4 Department of Zoology, University of Stuttgart, Stuttgart, Germany; Institut Européen de Chimie et Biologie, France

## Abstract

**Background:**

Tardigrades are small, multicellular invertebrates which are able to survive times of unfavourable environmental conditions using their well-known capability to undergo cryptobiosis at any stage of their life cycle. *Milnesium tardigradum* has become a powerful model system for the analysis of cryptobiosis. While some genetic information is already available for *Milnesium tardigradum* the proteome is still to be discovered.

**Principal Findings:**

Here we present to the best of our knowledge the first comprehensive study of *Milnesium tardigradum* on the protein level. To establish a proteome reference map we developed optimized protocols for protein extraction from tardigrades in the active state and for separation of proteins by high resolution two-dimensional gel electrophoresis. Since only limited sequence information of *M. tardigradum* on the genome and gene expression level is available to date in public databases we initiated in parallel a tardigrade EST sequencing project to allow for protein identification by electrospray ionization tandem mass spectrometry. 271 out of 606 analyzed protein spots could be identified by searching against the publicly available NCBInr database as well as our newly established tardigrade protein database corresponding to 144 unique proteins. Another 150 spots could be identified in the tardigrade clustered EST database corresponding to 36 unique contigs and ESTs. Proteins with annotated function were further categorized in more detail by their molecular function, biological process and cellular component. For the proteins of unknown function more information could be obtained by performing a protein domain annotation analysis. Our results include proteins like protein member of different heat shock protein families and LEA group 3, which might play important roles in surviving extreme conditions.

**Conclusions:**

The proteome reference map of *Milnesium tardigradum* provides the basis for further studies in order to identify and characterize the biochemical mechanisms of tolerance to extreme desiccation. The optimized proteomics workflow will enable application of sensitive quantification techniques to detect differences in protein expression, which are characteristic of the active and anhydrobiotic states of tardigrades.

## Introduction

**Figure 1:**
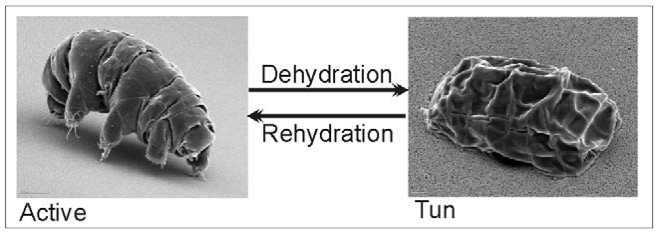
SEM images of *M. tardigradum* in the active and tun state. Tardigrades are in the active form when they are surrounded by at least a film of water. By loosing most of their free and bound water (>95%) anhydrobiosis occurs. Tardigrades begin to contract their bodies and change their body structure into a so-called tun.

Many organisms are exposed to unfavourable, stressful environmental conditions, either permanently or for just certain periods of their lives. To survive these extreme conditions, they possess different mechanisms. One of amazing adaptation is anhydrobiosis (from the Greek for “life without water”), which has puzzled scientists for more than 300 years. For the first time the Dutch microscopist Anton van Leeuwenhoek (1702) gave a formal description of this phenomenon. He reported the revival of “animalcules” from rehydrated moss samples. In extreme states of dehydration, anhydrobiotic invertebrates undergo a metabolic dormancy, in which metabolism decreases to a non-measurable level and life comes to a reversible standstill until activity is resumed under more favourable conditions [1]. One of the best known anhydrobiotic organisms are tardigrades. Tardigrades remain in their active form when they are surrounded by at least a film of water. By loosing most of their free and bound water (>95%) anhydrobiosis occurs [2]. Tardigrades begin to contract their bodies and change their body structure into a so-called tun state (Figure 1). In the dry state these organisms are highly resistant to environmental challenge and they may remain dormant for a long period of time. Based on their amazing capability to undergo anhydrobiosis, tardigrades colonise a diversity of extreme habitats [3], and they are able to tolerate harsh environmental conditions in any developmental state [4]. Possessing the ability to enter anhydrobiosis at any stage of life cycle, tardigrades can extend their lifespan significantly [4], [5]. Additionally, in the anhydrobiotic state, tardigrades are extraordinary tolerant to physical extremes including high and subzero temperatures [6], [7], [8], high pressure [6], [9], and extreme levels of ionizing radiation [10], [11]. Interestingly, tardigrades are even able to survive space vacuum (imposing extreme desiccation) and some specimens have even recovered after combined exposure to space vacuum and solar radiation [12].

Anhydrobiosis seems to be the result of dynamic processes and appears to be mediated by protective systems that prevent lethal damage and repair systems. However, the molecular mechanisms of these processes are still poorly understood. Up to now investigations of mechanisms of desiccation tolerance have focused mainly on sugar metabolisms, stress proteins and a family of hydrophilic proteins called LEA (late embryogenesis abundant). The presence of non-reducing trehalose and its expression during anhydrobiosis has been reported for different anhydrobiotic species [13], [14], which indicates the important role of trehalose in anhydrobiosis. However, the existence of anhydrobiotic animals that exhibit excellent desiccation tolerance without having disaccharides in their system [15], [16] shows that sugars alone do not sufficiently explain these phenomena.

*Milnesium tardigradum* Doyère (1840) is a very well known species of carnivorous tardigrade. Different aspects of the life history of this species have been already described [17]. While some genetic studies of *M. tardigradum* exist [18] almost nothing is known about the proteome. Partial sequences of three heat shock protein (hsp70 family) genes and the housekeeping gene beta-actin have been described [18] and the relation of hsp70 expression to desiccation tolerance could be shown by real time PCR [18] and by de novo protein synthesis [6]. Since no trehalose could be detected in *M. tardigradum* [19], investigating proteins and posttranslational modifications is of particular importance to clarify surviving mechanisms during desiccation.

To gain insight into the unique adaptation capabilities of tardigrades on the protein level we aimed to establish a comprehensive proteome reference map of active *M. tardigradum* employing optimized protocols for protein extraction, generation of high-resolution 2D gels and high-throughput protein identification by electrospray ionization tandem mass spectrometry (ESI-MS/MS). The proteome reference map of *M. tardigradum* provides the basis for further studies in order to understand important physiological processes such as anhydrobiosis and stress resistance. The optimized proteomics workflow will enable application of sensitive quantification techniques to detect differences in protein expression, which are characteristic of active and anhydrobiotic states. Thus, our proteomic approach together with in-depth bioinformatic analysis will certainly provide valuable information to solve the over 300 years existing puzzle of anhydrobiosis.

## Results

### Preparation of Protein Extracts from Active Tardigrades

**Figure 2:**
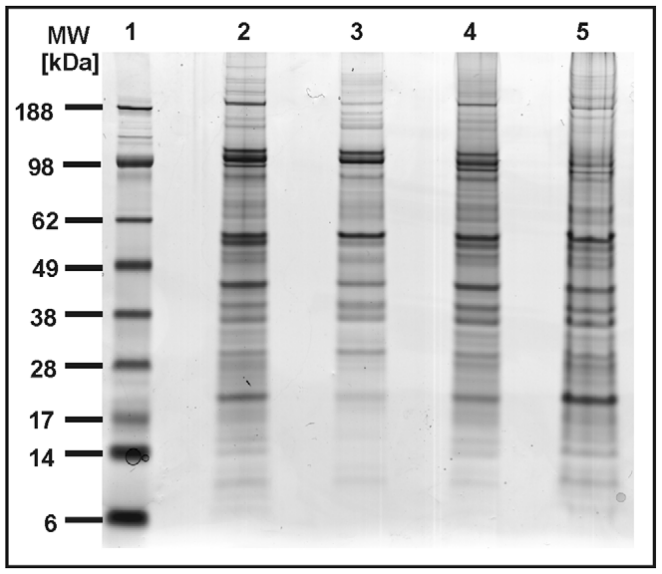
Comparison of different workup protocols for *M. tardigradum*. Total protein extract of tardigrades in the active state was separated on a one-dimensional polyacrylamide gel. Lane 1: Rainbow molecular weight marker. Lane 2: Protein extract of whole tardigrades without any precipitation step. Lane 3: Protein extract after TCA precipitation. Lane 4: Protein extract after chloroform/methanol precipitation. Lane 5: Protein extract using clean-up kit.

**Figure 3:**
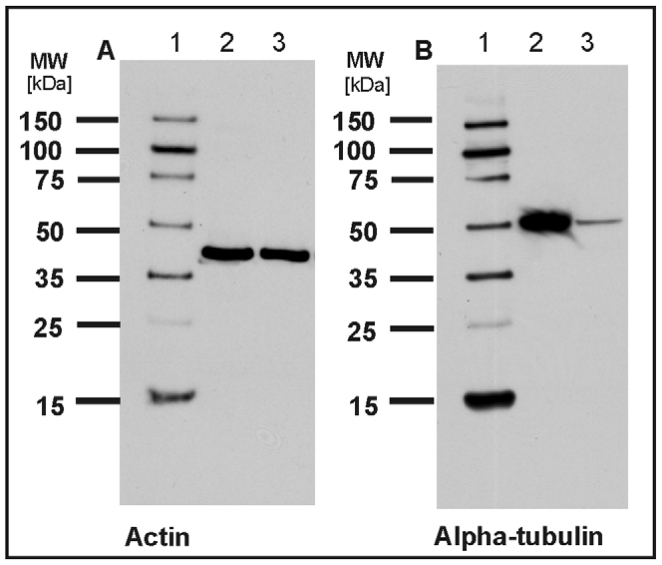
Analysis of protein degradation in total protein extracts of tardigrades by Western blot analysis. Actin (A) and alpha tubulin (B) were used as marker proteins for the detection of proteolysis. Lane 1A and 1B: DualVue Western blotting marker. Lane 2A and 2B: Total protein extract of HeLa cells as control. Lane 3A and 3B: Total protein extract of *M. tardigradum*. Notably, no protein degradation was observed during the workup procedure.

To establish and optimize a reliable and robust protocol for the extraction of proteins from tardigrades in the active state we applied different workup protocols and evaluated them by one-dimensional (1D) gel electrophoresis. Figure 2 shows the separation of protein extracts from whole tardigrades without any precipitation step (lane 2), after trichloroacetic acid/acetone precipitation (lane 3), after chloroform/methanol precipitation (lane 4) and after using a commercially available clean-up kit (lane 5). When using trichloroacetic acid/acetone precipitation we lost many proteins especially in the low molecular weight range. Chloroform/methanol precipitation and application of clean-up kit delivered satisfying results but also using the whole protein lysate directly without any further purification resulted in high yields across the entire molecular weight range. This workup protocol was therefore used throughout our proteome study. To evaluate the quality of our protocol especially with respect to proteolysis we performed Western blot analysis to detect any protein degradation. Since no proteins have been identified so far, we have chosen two polyclonal antibodies directed against the highly conserved proteins actin and alpha-tubulin. As shown in Figure 3A and 3B both proteins could be detected at their expected molecular weight at approx. 40 and 50 kDa, respectively, which is in agreement with the protein bands of the control lysate of HeLa cells. Importantly, no protein degradation could be observed during our sample preparation.

### Two Dimensional Gel Electrophoresis (2-DE)

**Figure 4:**
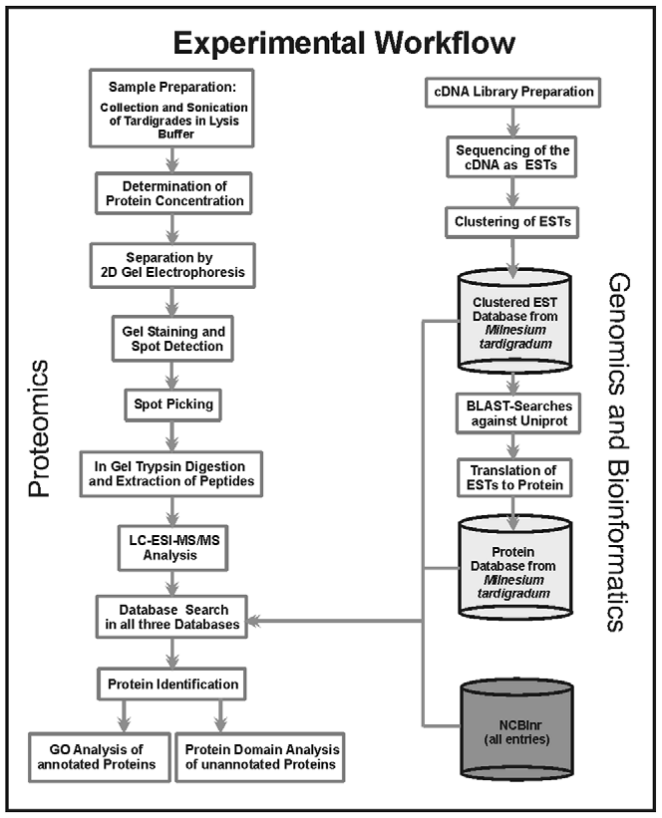
The experimental workflow to developing the proteome map. Tardigrades were sonicated directly in lysis buffer. Total protein extracts were separated by two-dimensional gel electrophoresis. After silver staining protein spots were picked and in-gel digested with trypsin. MS/MS data obtained by LC-ESI-MS/MS analysis were searched against the NCBInr database, the clustered tardigrade EST database and the tardigrade protein database. Identified proteins with annotation were classified in different functional groups using the Blast2GO program. Identified proteins without annotation were analysed with the DomainSweep program to annotate protein domains.

**Figure 5:**
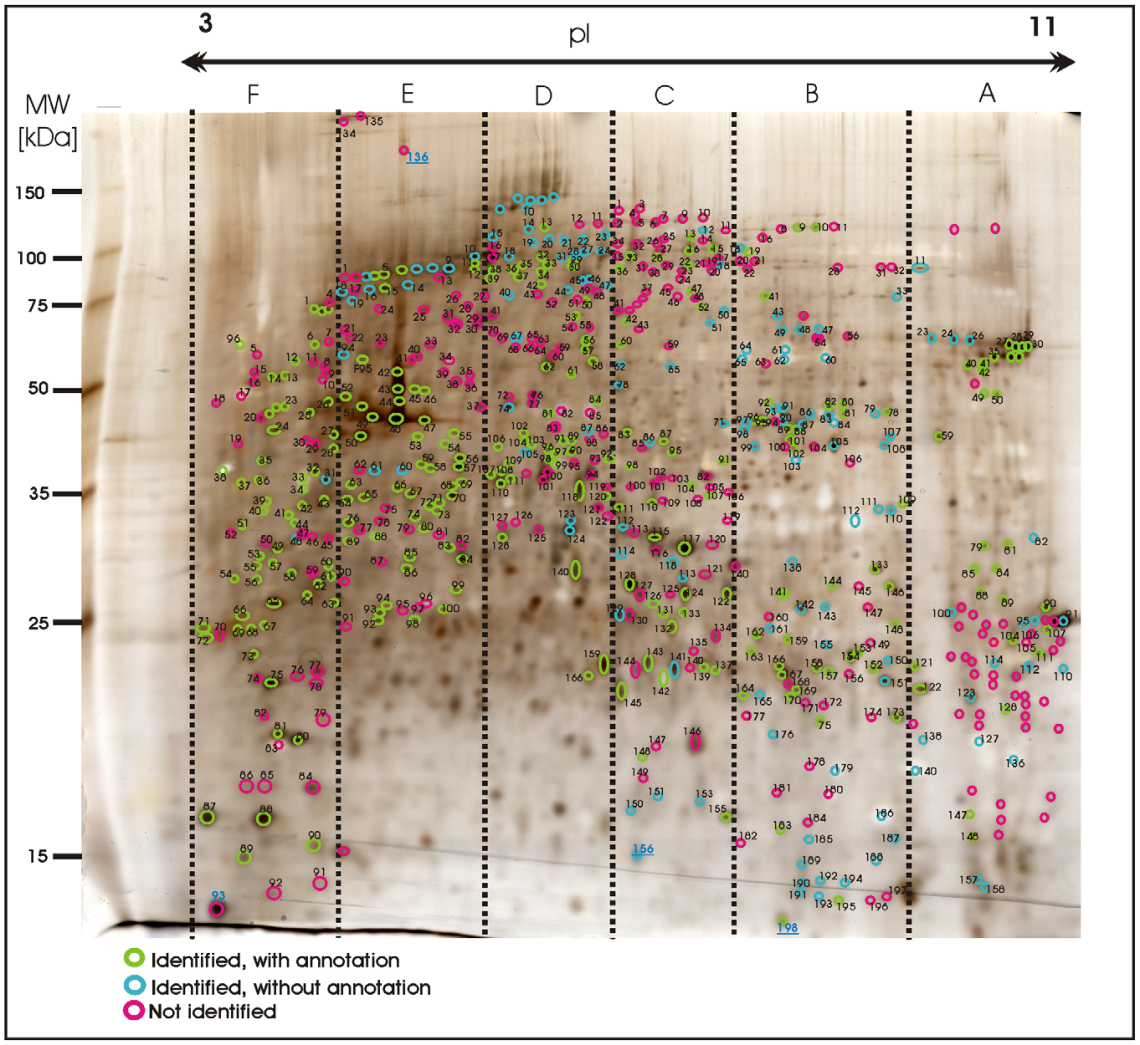
Image of a preparative 2D-gel with selected analysed protein spots. Total protein extract of 400 tardigrades in the active state corresponding to 330 µg was separated by high resolution two-dimensional gel electrophoresis. Proteins were visualised by silver staining. Three different categories are shown: Identified proteins with functional annotation are indicated in green, identified proteins without annotation are indicated in blue and not yet identified proteins are indicated in red.

The establishment of an optimized workup protocol was a prerequisite for high quality 2D gels from tardigrades in the active state. The proteomics workflow is depicted in Figure 4. One important step in the workflow is the collection and preparation of the samples. To avoid contamination with food-organisms, tardigrades were washed several times and starved over 3 days. Direct homogenization and sonication of deep-frozen tardigrades in ice cold lysis buffer without any previous precipitation step yielded protein extracts which were separated by high resolution 2D gel electrophoresis. For maximal resolution of protein spots and high loading capacity (330 µg proteins) we used pI 3–11 NL strips (24 cm) for the first dimension. Thus, high resolution separation could be achieved in the acidic as well as in the basic pH range as shown in the image of the silver stained preparative gel of whole protein extract (Figure 5).

Approximately 1000 protein spots were automatically detected on the 2D gel image using the Proteomweaver image software. A total of 606 protein spots were picked from the silver stained gel. These spots were digested with trypsin and after extraction of the tryptic peptides from the gel plugs peptide mixtures were analyzed by nanoLC-ESI-MS/MS.

### Protein Identification

Identification of proteins depends on the representation of the sequence or a close homologue in the database. Since almost no genome or EST sequences of *M. tardigradum* are available to date in public databases we initiated the tardigrade EST sequencing project as outlined in figure 4 (Mali et al, submitted data). A cDNA library was prepared from tardigrades in different states (active, inactive, transition states). The cDNAs were sequenced as ESTs and clustered. Thereby, we obtained a nucleotide database containing 818 contigs and 2500 singlets. cDNA sequencing and generation of ESTs are still ongoing, thus the sequence coverage of *M. tardigradum* in the database is incomplete.

**Figure 6:**
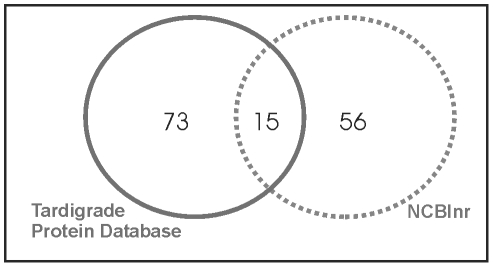
Comparison of database performance for protein identification. Protein spots were analysed by nanoLC-ESI-MS/MS and searched against the NCBInr database and the tardigrade protein database. The diagram illustrates the number of positive identifications in the respective database and the overlap between the two databases.

For protein identification we used the following databases: the database of *M. tardigradum* containing the clustered ESTs as outlined above, the tardigrade protein database, which was translated from the clustered EST database and thus represents a subdatabase containing only annotated proteins with known function and the publicly available NCBInr database. The selected 606 spots from the 2D gel correspond to some highly expressed proteins, but mostly to spots in the medium and low expression range. A total of 271 spots could be identified from the tardigrade protein and the NCBInr databases. Figure 6 demonstrates how identified proteins are distributed among these two databases. 56 unique proteins were successfully identified by searching the NCBInr database. It concerns proteins which are either highly conserved among different species e. g. actin or protein entries from *M. tardigradum* which are already available in the NCBInr database e.g. elongation factor 1-alpha. Further 73 unique proteins could be identified by searching the tardigrade protein database and another 15 unique proteins were present in both databases. Identical proteins that were identified from several spots were included only once in the statistics to avoid bias. Thus, the combination of the two databases was sufficient for the identification of 144 unique proteins. The corresponding protein spots are indicated by green circles in the 2D reference map shown in Figure 5. Table 1 shows an overview of identified proteins with annotation in different functional groups. In addition, detailed information about each of the identified 144 proteins including spot number, protein annotation, accession number (NCBInr and Tardigrade specific accession number), total protein score, number of matched peptides, peptide sequence and sequence coverage is listed in Table 2. The individual ion score is included in brackets at the end of every peptide sequence. Following ion scores indicate a significant hit (p<0.05): >53 for NCBInr searches, >14 for searches in the tardigrade protein database and >27 by searching the EST clustered database. Identical proteins identified in different spots are listed only once in Table 2. In these cases the spot with the highest protein score (in bold) is ranked at the top whereas the other spots are listed below. All further information such as accession numbers, peptide sequences and sequence coverage refer to the top-ranked spot.

**Table 1:** Overview of identified proteins classified in different functional groups.

Cytoskeleton elements and modulators	Enzymes	Proteases and protease inhibitors
Alpha-III tubulin	Glucan endo-1,3-beta-glucosidase	Cathepsin K
Beta-tubulin class-IV	Prostatic acid phosphatase	Cathepsin Z
Beta-tubulin class-I	Adenylate kinase isoenzyme 1	Cathepsin L1
Actin	Peptidyl-prolyl cis-trans isomerase	Neprilysin-2
Actin-5C	Glutamate dehydrogenase	Peptidase M17 precursor
Beta actin	Lysosomal acid phosphatase	Actinidain
Alpha actin	Mitochondrial malate dehydrogenase	Plasminogen
Actin, muscle-type (A2)	Arginine kinase	Aspartic protease inhibitor 8
muscle actin	Aconitase, mitochondrial	AFG3-like protein 2
Similar to alpha actinin CG4376-PB	Transaldolase	26S proteasome non-ATPase regulatory subunit 8
Myophilin	Aldolase A protein	Rab GDP dissociation inhibitor beta
Tropomyosin-1, isoforms 9A/A/B	Protein disulfide isomerase-3	Gamma-glutamyltranspeptidase
Tropomyosin	Matrix metalloproteinase-17	**Response to stress or heat**
Myosin regulatory light polypeptide 9	Mitochondrial long-chain enoyl-CoA hydratase/3-hydroxycyl-CoA	NADP-dependent isocitrate dehydrogenase
Myosin, essential light chain	Dehydrogenase alpha-subunit	Heat shock 70 kDa protein II
**Heat shock proteins**	Peroxidase	similar to heat shock cognate 70 protein isoform 2
Heat Shock Protein family member (hsp-3)	Methylmalonate-semialdehyde dehydrogenase	Short-chain dehydrogenase/reductase SDR YhdF
Heat Shock Protein family member (hsp-1)	Thioredoxin reductase 1	Aspartic protease inhibitor 8
Hsp 60	Succinyl-CoA ligase [GDP-forming] subunit beta, Mitochondrial E	UspA
Hsp 70	GTP-specific succinyl-CoA synthetase beta subunit	Rubber elongation factor protein (REF) (Allergen Hev b 1)
Heat shock cognate 70	Glycosyl transferase	Small rubber particle protein (SRPP) (22 kDa rubber particle protein)
Heat shock cognate 70 protein isoform 2	DEAD-box family (SNF2-like) helicase	Heat shock protein 90-beta
Heat shock 70 kDa protein II (HSP70 II)	Cysteine conjugate beta-lyase	Heat shock protein 83
Hsp 90-beta	26S proteasome non-ATPase regulatory subunit 13	Heat shock protein 60
Hsp90-alpha	GH19645	**Other Proteins**
Hsp90	**Glycolysis**	Translationally-controlled tumor protein homolog
Hsp 82	Glyceraldehyde-3-phosphate dehydrogenase	Elongation factor 1-alpha
Hsp 83	Triosephosphate isomerase	Elongation factor 1 gamma
Hsp108	Enolase	Elongation factor 2
Protein lethal(2)essential for life (member of Hsp20 family)	Phosphoglycerate kinase	Angiopoietin-related protein 1
**Embryonic/larval development**	**Transporters**	Spaghetti CG13570-PA
Vitellogenin-1	H(+)-transporting ATP synthase	Prohibitin
Vitellogenin-2	ATP synthase subunit d, mitochondrial	Proteasome subunit alpha type-4
Protein disulfide-isomerase 2	ATP synthase beta subunit	40S ribosomal protein S12
Heat Shock Protein family member (hsp-3)	Mitochondrial ATP synthase alpha subunit precursor	Periostin
Heat Shock Protein family member (hsp-1)	Annexin A6	Acetylcholine receptor subunit alpha-L1
Troponin C	**Antioxidant proteins**	Nucleosome remodelling factor – 38kD CG4634-PA
Putative LEA III protein isoform 2	Thiol-specific antioxidant protein	Coiled-coil domain-containing protein 25
GDP-Mannose Dehydratase	Superoxide dismutase [Cu-Zn]	Calreticulin
Tropomyosin	Peroxiredoxin-5, mitochondria	Lipoprotein-related protein
**Dormancy related protein**	Peroxiredoxin-4	14-3-3 protein beta/alpha-2 (Protein 14-3-3B2)
Putative LEA III protein isoform 2	Glutathione S-transferase	60S ribosomal protein L26-1
	Peroxiredoxin-6	Histone H4
		Histone H2B.2

Identified proteins with annotation are listed in 8 different groups with majority in protein enzymes. We also identified many heat shock proteins and proteins, which are involved in embryonic development, response to stress/heat and dormancy.

**Table 2:** Identified proteins with annotation.

Spot no.	Protein name	NCBInr Accession no. (°)	Tardigrade specific Accession no. (contig/EST) (∧)	Total protein score	No. of unique/significant peptides	MS/MS peptide sequence (Indv. Ion score)	Sequence coverage
A30*	elongation factor 1-alpha [Milnesium tardigradum]	gi|4530101	(EZ048811)	544	5	**K.YAWVLDK.L(23)**	**51%**
A27*, A28*, A29*, A32*,						**R.LPLQDVYK.I(52)**	
A33*, A35*, A40*, A41*,						**K.IGGIGTVPVGR.V(56)**	
A42*, A49*, A50*, A59*,						**R.EHALLAYTLGVK.Q(65)**	
A81*, A84∧, A85∧, A88*,						**K.YYVTIIDAPGHR.D(67)**	
A107∧, B78∧, B80*, B81*,						**K.MDSSEPPFSEDR.F + Oxidation (M)(72)**	
C28°, C36°, D120°						**R.NGYTPVLDCHTAHIACK.F(18)**	
						**K.MDSSEPPFSEDRFNEIVK.E(12)**	
						**K.TLLEALDSISPPARPTDKPLR.L(69)**	
						**R.VETGVIKPGMVVTFAPTGLTTEVK.S(34)**	
						**K.NMITGTSQADCAVLVTPAPPGEFEAGISK.N(16)**	
						**K.SGDAAIVNLIPTKPLCVEAFSEYPPLGR.-(45)**	
**A79**	DB:Swissprot Frame:3 orf:3 Homolog:Angiopoietin-		EZ048825	35	1	**R.VFYTSDVPDPNR.C(35)**	**5%**
	related protein 1 Evalue:1e-29 Bitscore:130						
**A84**	DB:Swissprot Frame:1 orf:8 Homolog:Cathepsin K		GH986829	58	1	**K.LSEEFVR.D(13)**	**16%**
A85	Evalue:1e-16 Bitscore:73.6					**R.WSDVTRPGCK.G(46)**	
**A85**	DB:Swissprot Frame:1 orf:7 Homolog:Actinidain		EZ048769	33	1	**R.NSWGPNWANK.G(33)**	**18%**
	Evalue:1e-11 Bitscore:70.5						
	DB:Swissprot Frame:3 orf:3 Homolog:Glucan endo-		EZ048807	28	1	**K.EMFSVNDSPNKR.L + Oxidation (M)(28)**	**5%**
	1,3-beta-glucosidase A1 Evalue:1e-23 Bitscore:110						
**A90**	DB:Trembl Frame:-3 orf:1 Homolog:GF11309		EZ048774	192	3	**R.GAVSCIDSFVNR.C(68)**	**20%**
	Evalue:1e-06 Bitscore:57.8					**R.FNPQQPASILQDR.K(74)**	
						**K.DSLSQTQFTELCTR.S(49)**	
	spaghetti CG13570-PA [Drosophila melanogaster]	gi|17864228		55	1	**K.ILGAGFDSDTFADLLR.T(55)**	**2%**
**A104**	DB:Swissprot Frame:2 orf:3 Homolog:Prostatic acid		GH986832	162	4	**R.YSSYLGPK.F(53)**	**48%**
	phosphatase Evalue:3e-24 Bitscore:112					**K.TVWNNELGQLTSK.G(56)**	
						**K.FSIPEVLIVSSAVER.A(37)**	
						**R.AVQSTLVNAAGLFTPSGDTIWNSGSSEIGK.T(17)**	
**A89**				200	4	**R.YSSYLGPK.F(44)**	**37%**
						**R.SPIFTFPTDPYGK.T(63)**	
						**K.FSIPEVLIVSSAVER.A(48)**	
						**K.GMQQMYQLGQYLSAR.Y + 2 Oxidation (M)(45)**	
A111*	prohibitin [Aedes aegypti]	gi|157131967	(EZ048795)	121	2	**K.FNASQLITQR.Q(54)**	**7%**
						**R.VLPSICNEVLK.G(67)**	
**A121**	mitochondrial ATP synthase alpha subunit precursor	gi|47551121		97	1	**R.VLSIGDGIAR.V(45)**	**3%**
	[Strongylocentrotus purpuratus]					**R.VVDALGTPIDGK.G(54)**	
	ZK829.4 [Caenorhabditis elegans]	gi|17544676		57	1	**K.CAVVDVPFGGAK.G(53)**	**5%**
						**K.GFLGPGVDVPAPDMGTGER.E + Oxidation (M)(4)**	
**A122**,	DB:Swissprot Frame:1 orf:3 Homolog:Glutathione S-		EZ048812	439	8	**K.LSQYIER.I(38)**	45%
B170, B175	transferase 1 Evalue:1e-39 Bitscore:164					**K.VDGIIDFFK.D(65)**	
						**K.QVAQSAAILR.F(65)**	
						**R.FNLSGKDEFEK.A(72)**	
						**K.FFSTDVHQYLK.T(42)**	
						**K.DMQSSMVTWYR.E(66)**	
						**R.FAFAYAGQQFEDNR.I(44)**	
						**K.EQMPFGQLPILEVDGK.Q + Oxidation (M)(47)**	
**B153**	DB:Swissprot Frame:3 orf:2 Homolog:Glutathione S-		EZ048805	260	7	**K.YILGNDVK.Y(19)**	**55%**
B154	transferase Evalue:5e-44 Bitscore:177					**R.YLLEYVGEK.Y(43)**	
						**K.SYDQFETQPK.W(31)**	
						**K.QYQNLADYHK.R(5)**	
						**R.LMYMSQDFEK.E + Oxidation (M)(32)**	
						**K.HYDMFSQFLGNK.K + Oxidation (M)(32)**	
						**K.LTQSTAIMHFLAR.K + Oxidation (M)(70)**	
						**K.QSLGLPFPNIPYYIDGNTK.L(2)**	
						**K.TEEEQQQCDMVEGALSDFR.Q + Oxidation (M)(25)**	
**B166**	DB:Swissprot Frame:2 orf:1 Homolog:Glutathione S-		EZ048770	213	6	**K.QYLLGSDIK.Y(25)**	**31%**
B158	transferase Evalue:3e-43 Bitscore:176					**R.YLLEYVGEK.Y(43)**	
						**K.LMYGSQDFEK.D + Oxidation (M)(23)**	
						**K.LTQSNAILHHLAR.K(63)**	
						**K.LMYGSQDFEKDK.S + Oxidation (M)(41)**	
						**K.SEEEQQQCDMIEGALHDFR.M + Oxidation (M)(18)**	
**B169**	DB:Swissprot Frame:1 orf:3 Homolog:Probable		GH986911	96	2	**R.LLFAAADQK.Y(48)**	**20%**
	glutathione S-transferase 9 Evalue:8e-24					**K.VLAQTTSIVR.Y(48)**	
	Bitscore:110						
**D166**	DB:Swissprot Frame:3 orf:3 Homolog:Glutathione S-		GH986673	48	2	**K.DMLVAMQR.W(14)**	**18%**
	transferase 1 Evalue:3e-30 Bitscore:131					**K.LKGEEIMDYMK.D(11)**	
						**K.DQTPYGQLPILEVDGMK.I(23)**	
**D159**	DB:Swissprot Frame:1 orf:3 Homolog:Probable		EZ048796	405	8	**R.IIFDENDK.S(56)**	**33%**
	glutathione S-transferase 6 Evalue:2e-34					**R.SFEQFFEK.Y(31)**	
	Bitscore:146					**R.IIFDENDKSK.G(43)**	
						**K.FTEATFPASLR.S(47)**	
						**R.KFTEATFPASLR.S(63)**	
						**R.LIFHGTGEDFEDVR.L(61)**	
						**R.TEEALADSVVDATNDILGDLIR.I(48)**	
						**K.SRTEEALADSVVDATNDILGDLIR.I(58)**	
**A128**	DB:Swissprot Frame:3 orf:2 Homolog:Myophilin		EZ048783	273	6	**R.NFSDEQLR.Q(35)**	**40%**
	Evalue:1e-33 Bitscore:143					**R.LANEIQPGSIR.K(43)**	
						**R.AAEVCEWVNK.I(38)**	
						**K.ILGENVLSTSGK.M(84)**	
						**R.QGETMISLQYGSNK.G(48)**	
						**K.QNLNAVVICLESLGR.K(25)**	
**A147**	DB:Swissprot Frame:3 orf:2 Homolog:Adenylate		EZ048787	19	1	**K.GFLIDGFPR.E(19)**	**4%**
	kinase isoenzyme 1 Evalue:6e-42 Bitscore:171						
**A148**	DB:Swissprot Frame:1 orf:1 Homolog:Peptidyl-prolyl		EZ048822	140	3	**K.TSKPVVIADCGQL.-(34)**	**25%**
	cis-trans isomerase Evalue:2e-75 Bitscore:282					**K.TSKPVVIADCGQL.-(59)**	
						**K.HVVFGQVTEGLDIVK.K(49)**	
**B19**	DB:Swissprot Frame:2 orf:1 Homolog:Elongation		GH986944	22	1	**R.VFSGTVQTGQK.V(22)**	**6%**
	factor 2 Evalue:1e-72 Bitscore:271						
**B41**	PREDICTED: similar to CG8036-PB, isoform B	gi|66503776		125	1	**K.LDSDLEGHPTPR.L(48)**	**3%**
	isoform 2 [Apis mellifera]					**R.KLDSDLEGHPTPR.L(53)**	
**B82**	glutamate dehydrogenase, short peptide [Drosophila	gi|458803		116	1	**K.IIAEAANGPTTPAADK.I(50)**	**9%**
	melanogaster]					**K.TFIVQGFGNVGLHTTR.Y(62)**	
**B88**	DB:Swissprot Frame:2 orf:1 Homolog:Vitellogenin-2		EZ048823	200	4	**K.VSMINLR.L(41)**	**14%**
B9, B10, B89, B95, B96,	Evalue:1e-14 Bitscore:81.6					**R.AEDEYEWSR.A(40)**	
C22, C36, C47, C83, C87,						**K.TIVVLPSIYYK.N(51)**	
C117, C122, C124						**K.IMVVLPGHSIEITAPQGR.T + Oxidation (M)(68)**	
**B92**	NADP-dependent isocitrate dehydrogenase [Homo	gi|3641398		69	0	**K.DIFQEIYDK.Q(42)**	**4%**
	sapiens]					**R.FKDIFQEIYDK.Q(16)**	
B95	DB:Swissprot Frame:1 orf:1-3		GH986689	17	1	**R.KDPEAMNETAK.W + Oxidation (M)(17)**	**6%**
	Homolog:Uncharacterized protein C3orf33 homolog						
	Evalue:3e-06 Bitscore:						
**B102**	DB:Swissprot Frame:3 orf:1 Homolog:Lysosomal		EZ048780	90	2	**K.FLEPVTVPR.A(52)**	**46%**
B101	acid phosphatase Evalue:3e-10 Bitscore:65.1					**K.FILYSAHDNTISALLAAFK.A(28)**	
						**K.NNPNNVFDAPTTVIFPGCSEFCPLDQLR.K(10)**	
**109**	mitochondrial malate dehydrogenase precursor	gi|33439518		218	3	**R.IQDAGTEVVNAK.A(64)**	**11%**
A121	[Branchiostoma belcheri tsingtaunese]					**R.DDLFNTNASIVR.D(66)**	
						**K.AGAGSATLSMAYAGAR.F(87)**	
**B146**	hypothetical protein TRIADDRAFT_63625	gi|195999922		342	4	**R.VEIIANDQGNR.I(38)**	**8%**
B141, B144	[Trichoplax adhaerens]					**R.ITPSYVAFTADGER.L(91)**	
						**R.IINEPTAAAIAYGLDK.K(79)**	
						**K.NQLTSNPENTVFDVK.R(72)**	
						**R.IINEPTAAAIAYGLDKK.E(62)**	
**B173**	heat shock cognate 70 [Aedes aegypti]	gi|94468966		235	2	**K.IQVEYKGETK.N(38)**	**8%**
						**K.MKETAEAYLGK.T + Oxidation (M)(62)**	
						**R.IINEPTAAAIAYGLDK.K(28)**	
						**K.STAGDTHLGGEDFDNR.L(50)**	
						**R.IINEPTAAAIAYGLDKK.T(57)**	
**C131**	Heat shock 70 kDa protein II (HSP70 II)	gi|123622		154	2	**K.ETAEAYLGK.E(34)**	**8%**
						**K.VEIIANDQGNR.T(60)**	
						**R.TTPSYVAFTDTER.L(60)**	
**C133**	PREDICTED: similar to heat shock cognate 70	gi|193603576		153	2	**K.VEIIANDQGNR.T(60)**	**8%**
	protein isoform 2 [Acyrthosiphon pisum]					**R.TTPSYVGFTDTER.L(62)**	
						**R.IINEPTAAAIAYGLDK.K(16)**	
						**K.STAGDTHLGGEDFDNR.M(16)**	
**B148**	DB:Swissprot Frame:3 orf:2 Homolog:Malate		GH986821	179	3	**R.AIGQMAIQLK.N(52)**	**24%**
	dehydrogenase, cytoplasmic Evalue:3e-66					**K.DQGSALNQYAK.K(60)**	
	Bitscore:251					**K.ILVVGNPANTNAYILSHYAPSLPK.E(67)**	
**B152**	H(+)-transporting ATP synthase [Rattus norvegicus]	gi|57029		92	1	**K.LELAQYR.E(31)**	**8%**
						**R.EAYPGDVFYLHSR.L(61)**	
B164*	ATPase subunit [Beta vulgaris subsp. Vulgaris]	gi|11263	(EZ048779)	64	0	**K.LELAQYR.E(34)**	**4%**
						**R.GIRPAINVGLSVSR.V(29)**	
B167*	DB:Swissprot Frame:1 orf:1 Homolog:Arginine	(gi|124264768)	EZ048827	254	6	**R.FLQAAQAVR.F(41)**	**33%**
B133∧, B157∧, B159*,	kinase Evalue:5e-90 Bitscore:295					**K.LIDDHFLFK.E(39)**	
B162∧, C91*, C98∧, C104∧,						**K.LNFPNPDPEGK.Y(60)**	
C107∧, C137∧, C142∧,						**R.KYMTPEIIQK.L + Oxidation (M)(29)**	
D98∧, D159∧						**R.SLQGFPFNPLLNEQQYK.E(30)**	
						**K.DLFYPIINDYHVGFDIEK.G(55)**	
**B183**	DB:Swissprot Frame:2 orf:5 Homolog:Peroxiredoxin-		EZ048816	92	1	**R.HLPSYVK.K(10)**	**24%**
	5, mitochondrial Evalue:3e-40 Bitscore:150					**K.VHLLADPR.G(9)**	
						**K.LNIEPDGTGVECSIADR.I(73)**	
**C28**	pre-mRNA binding K protein, hnRNP K [Xenopus	gi|299029		58	1	**R.ILSISADIETIGEILK.K(58)**	**4%**
C36	laevis, Peptide, 396 aa]						
**C42**	Heterogeneous nuclear ribonucleoprotein K [Mus	gi|13384620		67	0	**R.ITAVLSPR.I(43)**	**7%**
	musculus]					**K.ILLLLLSGAK.L(24)**	
**C47**	PREDICTED: similar to aconitase, mitochondrial	gi|156537745		58	0	**K.NTIVTSYNR.N(25)**	**2%**
	[Nasonia vitripennis]					**K.ILYSHLDEPQK.Q(33)**	
**C52**	peptidase M17 precursor [Clonorchis sinensis]	gi|118429525		55	1	**K.GITYDTGGADVK.A(55)**	**2%**
**C60**	DB:Swissprot Frame:2 orf:1 Homolog:Gamma-		GH986789	53	2	**K.DMSSPEQDLYHQR.F + Oxidation (M) (31)**	**11%**
	glutamyltranspeptidase 1 Evalue:6e-49 Bitscore:194					**K.LKEFLTSPQVAQSTR.R(22)**	
**C87**	GDP-Mannose Dehydratase family member (gmd-2)	gi|17507723		61	1	**K.FYQASTSELYGK.V(61)**	**3%**
	[Caenorhabditis elegans]						
**C95**	Short-chain dehydrogenase/reductase SDR YhdF	gi|52079424		110	1	**K.GAIVAFTR.S(51)**	**7%**
	[Bacillus licheniformis ATCC 14580]					**K.TAIITGGDSGIGR.A(59)**	
	DB:Swissprot Frame:2 orf:1		(GH986692)	31	1	**K.TALITGASTGIGR.A(31)**	**6%**
	Homolog:Uncharacterized oxidoreductase yhdF						
	Evalue:3e-28 Bitscore:125						
**C98**	DB:Swissprot Frame:3 orf:5 Homolog:Protein		EZ048820	59	2	**R.GYRPEEVTLK.T(15)**	**30%**
	lethal(2)essential for life Evalue:2e-11 Bitscore:70.9					**K.DGVLSVECPLPQGNR.L(44)**	
	DB:Swissprot Frame:-1 orf:1 Homolog:Probable					**K.TVVMGASFR.N + Oxidation (M)(11)**	
	transaldolase Evalue:6e-34 Bitscore:144		GH986571	35	1	**K.LLEELANSTAK.V(24)**	**18%**
**C110**	aldolase A protein [Homo sapiens]	gi|28595		71	1	**K.GILAADESTGSIAK.R(71)**	**12%**
**C111**	DB:Swissprot Frame:2 orf:1		GH986712	344	7	**K.ADVKEQDGQLSINGK.L(63)**	**51%**
	Homolog:Glyceraldehyde-3-phosphate					**K.DVDVVAINDPFIDIK.Y(49)**	
	dehydrogenase Evalue:6e-77 Bitscore:2					**K.FGIVEGLMTTVHAFTATQK.V + Oxidation (M)(39)**	
						**K.TMDIVSNASCTTNCLAPLAK.V(77)**	
						**R.AAIDKDVDVVAINDPFIDIK.Y(47)**	
						**K.VIISAPSADAPMFVCGVNLDK.Y(33)**	
						**K.VIISAPSADAPMFVCGVNLDKYDAK.T(35)**	
**C115**	DB:Swissprot Frame:1 orf:6 Homolog:Plasminogen		EZ048798	63	2	**K.GDFDEFIR.I(34)**	**19%**
	Evalue:6e-36 Bitscore:84.3					**R.AYSGGISADMLCGAAPGK.D(29)**	
**D159**				121	2	**R.GCAQPNYPGVYGR.M (46)**	**21%**
						**K.DSCQGDSGGPLVFLK.N(75)**	
**C126**	DB:Swissprot Frame:2 orf:4 Homolog:Proteasome		GH986859	47	1	**R.TTIFSPEGR.L(47)**	**4%**
	subunit alpha type-4 Evalue:4e-81 Bitscore:300						
**C128**	expressed hypothetical protein [Trichoplax	gi|196010133		105	1	**K.VGASEATLLNMLK.V(105)**	**4%**
	adhaerens]						
	F25H2.10 [Caenorhabditis elegans]	gi|17506815		97	1	**K.TSFFQALQIPTK.I(97)**	**3%**
	DEAD-box family (SNF2-like) helicase, putative	gi|84996109		54	1	**K.MLELISNIIK.K(54)**	**0%**
	[Theileria annulata]						
	ResB family protein [Hydrogenobaculum sp.	gi|195953863		54	1	**K.MLELISNIIK.K(54)**	**1%**
	Y04AAS1]						
**C132**	DB:Swissprot Frame:1 orf:1 Homolog:Peroxiredoxin-		EZ048818	393	8	**R.GLFIIDK.K(28)**	**31%**
	4 Evalue:4e-86 Bitscore:318					**R.GLFIIDKK.G(32)**	
						**K.TQIGKPAPDFK.G(27)**	
						**K.FENVNLSDYK.G(57)**	
						**R.QITMNDLPVGR.S(51)**	
						**K.GKFENVNLSDYK.G(59)**	
						**R.CNVYGSGDVYPER.S(58)**	
						**K.DYGVYLEDAGHTLR.G(81)**	
**C139**	DB:Swissprot Frame:1 orf:1 Homolog:ATP synthase		EZ048797	81	2	**K.VLAFPESPAK.I(33)**	**13%**
D166	subunit d, mitochondrial Evalue:4e-27 Bitscore:121					**R.VPVPGLVDQFR.K(48)**	
**C143**	Glyceraldehydes-3-phosphate dehydrogenase	gi|7274154		107	1	**R.VPVPDVSVVDLTVR.L(107)**	**4%**
C145∧	[Achlya bisexualis]						
C143*	DB:Swissprot Frame:3 orf:2-4	(gi|1351273)	GH986530	281	5	**K.AIADVISDWSK.V(67)**	**33%**
B163∧	Homolog:Triosephosphate isomerase BEvalue:4e-					**R.EGNQTETVVFR.Q(47)**	
	69 Bitscore:260					**K.DVGAEWVILGHSER.R(82)**	
						**K.VVIAYEPVWAIGTGK.T(47)**	
						**K.EASGAFTGEISPAMLK.D(38)**	
**C145**	DB:Swissprot Frame:2 orf:3 Homolog:Peroxiredoxin-		EZ048781	113	3	**K.IGSPAPDFK.A(41)**	**19%**
						**R.GLFIIDQK.G(37)**	
	4 Evalue:4e-65 Bitscore:247					**K.AVAVIDGQFQDIQLSTLK.G(35)**	
	thiol-specific antioxidant protein [Homo sapiens]	gi|438069		54	1	**R.QITVNDLPVGR.S**	**5%**
**C148**	RecName: Full = Aspartic protease inhibitor 8	gi|124012		60	0	**R.GALGGDVYLGK.S(12)**	**10%**
						**R.GALGGDVYLGK.S(48)**	
**C155**	DB:Swissprot Frame:1 orf:1 Homolog:Superoxide		GH986811	401	6	**R.VTSAVAVMK.G (45)**	**33%**
	dismutase [Cu-Zn] Evalue:2e-48 Bitscore:192					**R.LACGIVGVVGGTK.-(69)**	
						**R.VTSAVAVMKGDSPVK.A + Oxidation (M)(32)**	
						**R.GLPAAESKIHGNSGGR.L(70)**	
						**R.HVGDLGNLVADASGTAK.I(137)**	
						**K.IDITDSLMSLMGEHSIVGR.A + 2 Oxidation (M)(48)**	
**D13**	DB:Swissprot Frame:1 orf:1 Homolog:40S ribosomal		GH986534	22	1	**K.LDADSLPR.K(22)**	**6%**
	protein S12 Evalue:2e-34 Bitscore:144						
**D53**	PREDICTED: similar to alpha actinin CG4376-PB	gi|91080533		120	1	**R.VGWEQLLTSINR.N(47)**	**3%**
	[Tribolium castaneum]					**R.NINEVENQILTR.D(58)**	
**D56**	DB:Swissprot Frame:1 orf:3 Homolog:Periostin		EZ048782	22	1	**K.QTEGETVFIPDDAAFGK.M(22)**	**6%**
	Evalue:1e-10 Bitscore:67.8						
**D57**	protein disulfide isomerase-3 [Haemaphysalis	gi|148717319		68	0	**K.HGVSGYPTLK.I(48)**	**3%**
	longicornis]						
**D61**	Enolase (2-phosphoglycerate dehydratase) (2-	gi|1169533		94	1	**R.GNPTVEVEVTTDK.G(74)**	**6%**
D109	phospho-D-glycerate hydro-lyase)					**K.VKIGMDVASSEFYK.D + Oxidation (M)(20)**	
**D61**	DB:Swissprot Frame:1 orf:1 Homolog:Matrix		GH986535	17	1	**R.FEVAEGFPK.S(17)**	**16%**
	metalloproteinase-17 Evalue:2e-06 Bitscore:50.8						
**D62**	DB:Trembl Frame:1 orf:1 Homolog:Vitellogenin 1		EZ048784	477	9	**K.FGNNIGQNIEK.Y(46)**	**34%**
B19, C13, C15, C16, C28,	Evalue:2e-05 Bitscore:53.1					**K.VLFDGNYVEIK.A(59)**	
C33, D29, D30, (D32-D39),						**K.KFGNNIGQNIEK.Y(75)**	
D42, D50, D58, E11, E12,						**K.EPILAIVSPVTGLK.V(74)**	
E15, E45, E54, E65, E66,						**R.AYLLQEGSCNAQIPQDK.K(42)**	
E73, F26						**R.AYLLQEGSCNAQIPQDKK.V(36)**	
						**R.DELFAVLAANANPSASPLEIR.R(75)**	
						**K.VSEYTILYNGQPIPQPPTEGK.F(22)**	
						**-.DNSRDELFAVLAANANPSASPLEIR.R(48)**	
**D81**	DB:Swissprot Frame:2 orf:1 Homolog:Actin-1		GH986913	33	1	**K.EISALAPNTIK.-(33)**	**5%**
	Evalue:6e-87 Bitscore:319						
**D84**	UspA [Bacillus coagulans 36D1]	gi|124521548		56	1	**R.ILVAIDGSK.M(56)**	**6%**
							
	DB:Swissprot Frame:3 orf:1 Homolog:Aldehyde		EZ048791	160	4	**K.ALYLSQGIR.A(38)**	**37%**
	dehydrogenase, mitochondrial Evalue:2e-47					**K.YGLAASVMTK.D + Oxidation (M)(22)**	
	Bitscore:188					**-.GYFIEPTVFADVK.D(48)**	
						**R.ELGEYGLDAYTEVK.T(53)**	
**D90**	DB:Swissprot Frame:2 orf:1 Homolog:Annexin A6		EZ048803	223	6	**K.DLFDDLKK.E(24)**	**48%**
D89, D96	Evalue:1e-37 Bitscore:143					**R.DHYNPTIR.A(21)**	
						**K.GIGTDEDTVIK.I(44)**	
						**R.HLLFAIITTR.R(47)**	
						**R.AFQPFNPDNDAK.A(38)**	
						**R.EVIDDIVSDTSGYFR.H(43)**	
						**K.AIAGAGTSEEDLIEIMLTR.N + Oxidation (M)(6)**	
D91*	elongation factor 1 gamma [Bombyx mori]	gi|112983898	(EZ048793)	60	1	**K.VPAFESADGK.V(58)**	**2%**
D104°, D106°							
**D92**	mitochondrial long-chain enoyl-CoA hydratase/3-	gi|510108		57	0	**K.ALTSFER.D(7)**	**3%**
	hydroxycyl-CoA dehydrogenase alpha-subunit					**K.DGPGFYTTR.C(34)**	
	[Rattus n					**K.VIGMHYFSPVDK.M(16)**	
	DB:Swissprot Frame:3 orf:1 Homolog:Peroxidase		EZ048773	30	1	**R.TGFTTDQMAILK.K + Oxidation (M)(30)**	**7%**
	Evalue:7e-19 Bitscore:94.0						
**D96**	cysteine conjugate beta-lyase [Takifugu rubripes]	gi|5002565		84	1	**K.ALVINTPNNPLGK.V(84)**	**3%**
**D96**	DB:Swissprot Frame:3 orf:5 Homolog:26S		GH986860	100	3	**K.LLEEVEK.K(12)**	**15%**
	proteasome non-ATPase regulatory subunit 13					**K.KLLEEVEK.K(26)**	
	Evalue:1e-29 Bit					**R.SAGGMSELYK.N(32)**	
						**R.LHGTYAEYFR.E(31)**	
**D103**	DB:Swissprot Frame:1 orf:1 Homolog:Neprilysin-2		EZ048772	32	1	**K.IIAQYSNFR.Y(32)**	**6%**
	Evalue:9e-31 Bitscore:133						
**D107**	Tubulin alpha-3 chain (Alpha-III tubulin)	gi|3915094		252	2	**R.LSVDYGK.K(27)**	**18%**
						**R.QLFHPEQLITGK.E(31)**	
						**R.LIGQIVSSITASLR.F(29)**	
						**R.AVFVDLEPTVIDEIR.T(64)**	
						**R.NLDIERPTYTNLNR.L(81)**	
						**R.FDGALNVDLTEFQTNLVPYPR.I(21)**	
**D110**	Tubulin beta-3 chain (Beta-tubulin class-IV)	gi|135464		152	1	**R.FPGQLNADLR.K(70)**	**7%**
						**K.LAVNMVPFPR.L(36)**	
						**R.AVLVDLEPGTMDSVR.S(46)**	
**D111**	GH19645 [Drosophila grimshawi]	gi|195053606		120	2	**K.KGIDAEVINLR.S(56)**	**7%**
						**R.VFLLGEEVAQYDGAYK.V(64)**	
**D118**	DB:Swissprot Frame:1 orf:4 Homolog:Histone H4		GH986770	18	1	**R.ISGLIYEETR.G(18)**	**12%**
	Evalue:9e-39 Bitscore:160						
	DB:Swissprot Frame:-3 orf:1 Homolog:Histone		EZ048778	15	1	**K.LILPGELAK.H(15)**	**9%**
	H2B.2 Evalue:8e-32 Bitscore:135						
**D140**	DB:Swissprot Frame:2 orf:3 Homolog:Acetylcholine		EZ048771	381	7	**K.LGSWTFAK.D(51)**	**23%**
D128	receptor subunit alpha-L1 Evalue:1e-16 Bitscore:87					**R.LQYTDSAVK.K(34)**	
						**R.LQYTDSAVKK.I(34)**	
						**K.DELDVQTSQSK.F(68)**	
						**R.AFLSLNWQDHR.L(80)**	
						**K.FDDYFQSSVWK.F(61)**	
						**K.LGSWTFAKDELDVQTSQSK.F(53)**	
**D159**	DB:Swissprot Frame:2 orf:1 Homolog:Peroxiredoxin-		GH986904	403	8	**K.LAPEFEK.R(38)**	**53%**
	6 Evalue:8e-61 Bitscore:233					**R.NFDELLR.V(27)**	
						**R.VLDSLQLVSK.H(63)**	
						**K.HSVVTPVDWK.-(69)**	
						**K.LVLIYPATSGR.N(50)**	
						**K.DLESYCGMGGGK.F + Oxidation (M)(48)**	
						**K.MIALSCDDAQSHQGWIK.D + Oxidation (M)(40)**	
						**K.FGMLDPDELNSNNMPVTAR.A + Oxidation (M)(68)**	
**E4**	phosphoglycerate kinase[Verrucomicrobiae	gi|161075769		54	1	**K.AIGFLMEKELK.Y + Oxidation (M)(54)**	**2%**
	bacterium V4]						
**E5**	Rubber elongation factor protein (REF) (Allergen	gi|132270		104	1	**R.SLASSLPGQTK.I(33)**	**18%**
D99	Hev b 1)					**K.FVDSTVVASVTIIDR.S(71)**	
**E5**	Small rubber particle protein (SRPP) (22 kDa rubber	gi|14423933		87	0	**K.AEQYAVITWR.A(43)**	**14%**
	particle protein) (22 kDa RPP) (Latex allergen					**R.IVLDVASSVFNTGVQEGAK.A(44)**	
	DB:Swissprot Frame:2 orf:1 Homolog:Liver						
	carboxylesterase Evalue:2e-33 Bitscore:142		EZ048809	21	1	**K.AIVVAVNYR.V(21)**	**5%**
E43*	actin [Heliothis virescens]	gi|14010639	(EZ048826)	667	7	**K.EITALAPSTMK.I(41)**	41%
						**R.AVFPSIVGRPR.H(73)**	
						**K.IWHHTFYNELR.V(73)**	
						**K.QEYDESGPSIVHR.K(94)**	
						**K.SYELPDGQVITIGNER.F(79)**	
						**R.VAPEEHPVLLTEAPLNPK.A(90)**	
						**K.YPIEHGIITNWDDMEK.I(56)**	
						**K.DLYANTVLSGGTTMYPGIADR.M(45) R.KDLYANTVLSGGTTMYPGIADR.M+Oxidation (M)(36)**	
						**R.TTGIVLDSGDGVSHTVPIYEGYALPHAILR.L(55)**	
E43*	DB:Swissprot Frame:1 orf:2 Homolog:Actin-5C		EZ048826	471	9	**R.DLTDYLMK.I(25)**	**37%**
D99, D106, D108, E71,	Evalue:7e-155 Bitscore:547					**R.GYSFVTTAER.E(38)**	
E72, E84, E92, E94, E99,						**K.EITALAPSTMK.I(41)**	
E100, F44, F58, F61, F95						**K.AEYDESGPSIVHR.K(112)**	
						**K.SYELPDGQVITIGNER.F(79)**	
						**K.DLYANTVLSGGTTMYPGIADR.M(45)**	
						**R.KDLYANTVLSGGTTMYPGIADR.M+Oxidation (M)(36)**	
						**K.LCYVALDFEQEMATAAASSSLEK.S(39)**	
						**R.TTGIVLDSGDGVSHTVPIYEGYALPHAILR.L(55)**	
E47*	Actin, muscle-type (A2)	gi|3121741	(EZ048826)	519	6	**K.RGILTLK.Y(23)**	30%
F64*, F95°						**K.AGFAGDDAPR.A(62)**	
						**R.DLTDYLMK.I(24)**	
						**R.GYSFVTTAER.E(40)**	
						**K.EITALAPSTMK.I + Oxidation (M)(40)**	
						**R.AVFPSIVGRPR.H(59)**	
						**K.IWHHTFYNELR.V(60)**	
						**K.QEYDESGPSIVHR.K(58)**	
						**K.SYELPDGQVITIGNER.F(79)**	
						**R.VAPEEHPVLLTEAPLNPK.A(75)**	
E48*	actin 5C [Lycosa singoriensis]	gi|161661023	(EZ048826)	644	6	**K.AGFAGDDAPR.A(80)**	35%
E44*						**R.DLTDYLMK.I + Oxidation (M)(15)**	
						**R.GYSFVTTAER.E(44)**	
						**R.AVFPSIVGRPR.H(98)**	
						**K.IWHHTFYNELR.V(66)**	
						**K.QEYDESGPSIVHR.K(100)**	
						**K.SYELPDGQVITIGNER.F(70)**	
						**R.VAPEEHPVLLTEAPLNPK.A(83)**	
						**K.YPIEHGIITNWDDMEK.I + Oxidation (M)(25)**	
						**K.DLYANTVLSGGTTMYPGIADR.M(47)**	
						**R.KDLYANTVLSGGTTMYPGIADR.M+Oxidation (M)(18)**	
E50*	beta-actin [Rachycentron canadum]	gi|161376754	(EZ048826)	501	4	**K.AGFAGDDAPR.A(59)**	33%
E6*, E42*, E49*, F27*,						**K.EITALAPSTMK.I(49)**	
F28*						**R.AVFPSIVGRPR.H(73)**	
						**K.IWHHTFYNELR.V(46)**	
						**K.SYELPDGQVITIGNER.F(79)**	
						**M.EEEIAALVVDNGSGMCK.A(50)**	
						**R.VAPEEHPVLLTEAPLNPK.A(43)**	
						**K.DLYANTVLSGGTTMYPGIADR.M(24)**	
						**R.KDLYANTVLSGGTTMYPGIADR.M+Oxidation (M)(53)**	
E52*	alpha-actin (aa 40-375) [Mus musculus]	gi|49864	(EZ048826)	106	0	**R.GYSFVTTAER.E(34)**	11%
E85*, D103*						**K.EITALAPSTMK.I + Oxidation (M)(22)**	
						**K.SYELPDGQVITIGNER.F(50)**	
**E57**	muscle actin	gi|797290		290	1	**R.DLTDYLMK.I (24)**	25%
D104*, D97*, E69*,						**R.GYSFVTTAER.E(43)**	
E68*,E67*, E59*						**K.EITALAPSTMK.I(49)**	
E58*, E55*, E53*, E93°						**K.IWHHTFYNELR.V(39)**	
						**K.QEYDESGPSIVHR.K(58)**	
						**R.VAPEEHPVLLTEAPLNPK.A(39)**	
						**K.YPIEHGIITNWDDMEK.I(19)**	
**E63**	DB:Swissprot Frame:1 orf:1 Homolog:AFG3-like		GH986706	35	1	**K.CFELLSEK.K(9)**	**12%**
	protein 2 Evalue:9e-58 Bitscore:221					**K.GLGYAQYLPR.E(27)**	
**E64**	heat shock protein 90 alpha [Fundulus heteroclitus	gi|77999578		62	1	**R.FYTSASGDEMVSLK.D+ Oxidation (M)(62)**	**6%**
	macrolepidotus]						
E70*	actin [Paraphidippus aurantius]	gi|167683068	(EZ048826)	376	2	**R.DLTDYLMK.I(15)**	42%
D102*, E51*, E56*						**R.GYSFVTTAER.E(41)**	
						**K.EITALAPSTMK.I(46)**	
						**K.IWHHTFYNELR.V(40)**	
						**K.SYELPDGQVITIGNER.F(67)**	
						**R.VAPEEHPVLLTEAPLNPK.A(59)**	
						**K.YPIEHGIITNWDDMEK.I(8)**	
						**R.TTGIVLDSGDGVSHTVPIYEGYALPHAILR.L(16)**	
**E73**	PREDICTED: similar to Nucleosome remodelling	gi|66507623		85	1	**K.GDNDPIDVLEIGYK.V(85)**	**1%**
	factor – 38kD CG4634-PA [Apis mellifera]						
**E74**	DB:Swissprot Frame:1 orf:4 Homolog:Protein		GH986548	118	3	**K.SLAPEYAK.A(16)**	**17%**
E76, E88, F62	disulfide-isomerase Evalue:2e-44 Bitscore:178					**K.DNFEDALK.E(21)**	
						**K.VDATVETDLATK.Y(80)**	
**E80**	DB:Swissprot Frame:1 orf:3-5 Homolog:Cathepsin Z		GH986945	102	2	**K.VGDFGPISGR.E(47)**	**11%**
	Evalue:1e-63 Bitscore:243					**K.TFNQCGTCSEFGK.C(55)**	
**E83**	DB:Swissprot Frame:3 orf:1 Homolog:26S		EZ048799	154	4	**K.DLIPDSSLR.T(40)**	**23%**
	proteasome non-ATPase regulatory subunit 8					**R.IYYYDWK.D(5)**	
	Evalue:4e-48 Bits					**K.CEALLNQIK.V(21)**	
						**R.DVLEMGAQLAILK.R(45)**	
						**R.ACPEVNLNSLCR.M(44)**	
**E86**	DB:Swissprot Frame:3 orf:4 Homolog:Coiled-coil		EZ048808	21	1	**K.LSSAHVYLR.L(21)**	**6%**
	domain-containing protein 25 Evalue:2e-46						
	Bitscore:1						
**E89**	ATP synthase beta subunit [Asteria miniata]	gi|46909233		346	3	**K.AHGGYSVFAGVGER.T(33)**	**24%**
D140						**R.FTQAGSEVSALLGR.I(97)**	
						**R.VALTGLTVAEYFR.D(84)**	
						**K.TVLIMELINNVAK.A(62)**	
						**K.VALVYGQMNEPPGAR.A + Oxidation (M)(38)**	
						**R.GIAELGIYPAVDPLDSTSR.I(26)**	
						**R.EGNDLYHEMIEGGVISLK.D + Oxidation (M)(7)**	
**E46**				191	0	**K.IGLFGGAGVGK.T(41)**	**13%**
						**R.IPVGPETLGR.I(34)**	
						**K.VVDLLAPYAK.G(40)**	
						**R.TIAMDGTEGLIR.G + Oxidation (M)(44)**	
						**R.FTQAGSEVSALLGR.I(33)**	
**E89**	DB:Swissprot Frame:2 orf:1 Homolog:Rab GDP		GH986887	152	3	**K.VALELLGPIR.Q(54)**	**45%**
	dissociation inhibitor beta Evalue:4e-51 Bitscore:201					**R.GTGQVFDFTK.V(55)**	
						**R.CICLLDHPIPNTK.D(6)**	
						**K.DALSTQIIIPQNQVNR.N(33)**	
						**R.NNDIYISVVSYTHQVAAK.G(4)**	
**E92**	DB:Swissprot Frame:1 orf:1		GH986892	75	2	**K.TVTSLWR.E(22)**	**11%**
E93	Homolog:Methylmalonate-semialdehyde					**R.ASFAGDMNFYGK.A(54)**	
	dehydrogenase [acylating], mitochond						
**E98**	DB:Swissprot Frame:3 orf:1 Homolog:Thioredoxin		GH986518	50	1	**R.TACTAEIGLDK.V(50)**	**5%**
	reductase 1, cytoplasmic Evalue:2e-73 Bitscore:275						
**F3**	DB:Swissprot Frame:2 orf:1 Homolog:Protein		EZ048794	527	9	**R.IDSFPTIK.L(43)**	**56%**
F1, F2, F27, F43, F49	disulfide-isomerase 2 Evalue:3e-64 Bitscore:244					**R.ITEFFGLTK.D(57)**	
						**K.NFDEVVMDK.S(56)**	
						**R.LISLADQLVK.Y(46)**	
						**K.GDNTVVEYGGER.T(38)**	
						**K.MDATANELEHTR.I + Oxidation (M)(72)**	
						**K.KGDNTVVEYGGER.T(62)**	
						**K.LSPIYDELGDHFK.D(79)**	
						**K.YKPEAGDLNPETLTK.F(64)**	
						**K.LKPHLNSQDVPEDWNAK.S(10)**	
**F6**	Tubulin beta-1 chain (Beta-tubulin class-I)	gi|57429		54	0	**R.YLTVAAIFR.G(15)**	**4%**
						**R.FPGQLNADLR.K(39)**	
**F12**	hsp 108 [Gallus gallus]	gi|63509		84	0	**R.ELISNASDALDK.I(50)**	**4%**
						**K.GVVDSDDLPLNVSR.E(31)**	
**F13**	hsp 82 [Drosophila pseudoobscura]	gi|9069		77	1	**R.ELISNASDALDK.I(77)**	**3%**
F14*	DB:Swissprot Frame:3 orf:2 Homolog:Calreticulin	(gi|195107681)	GH986835	365	7	**K.FVWTAGK.F(23)**	**40%**
B195∧, B198∧	Evalue:2e-69 Bitscore:262					**K.FYGDADLNK.G(42)**	
						**K.VFPSTMDQK.D(48)**	
						**R.FYGLSAAFPK.F(41)**	
						**K.DKPLVIQFSVK.H(59)**	
						**K.HEQNIDCGGGYVK.V(88)**	
						**K.EQFLDNKWEDR.W(65)**	
**F38**	DB:Swissprot Frame:2 orf:1 Homolog:Calreticulin		GH986920	159	3	**R.EIPNPAYK.G(19)**	**47%**
	Evalue:4e-19 Bitscore:94.7					**K.AAEDFANDTWGK.T(85)**	
						**K.SGTIFDDIIITDDIK.A(54)**	
**F21**	Heat Shock Protein family member (hsp-3)	gi|17568549		180	0	**R.LSPEDIER.M(39)**	**6%**
F22, F23	[Caenorhabditis elegans]					**K.FDLTGLPPAPR.G(43)**	
						**K.FEELNMDLFR.A(47)**	
**F24**	heat shock protein 90-beta [Danio rerio]	gi|18858875		83	0	**K.HFSVEGQLEFR.A(42)**	**5%**
						**K.EKYIDQEELNK.T(16)**	
	heat shock protein 90 [Danio rerio]	gi|555574		62	1	**K.ADLMNNLGTIAK.S + Oxidation (M)(62)**	**11%**
**F24**	DB:Swissprot Frame:2 orf:3 Homolog:Heat shock		EZ048788	214	3	**R.YMSLTDPK.Q + Oxidation (M)(6)**	**21%**
F25	protein 83 Evalue:1e-84 Bitscore:312					**K.ADMINNLGTIAK.S(73)**	
						**K.EDQMDYVEEK.K(47)**	
						**R.ELISNSSDALDK.I(89)**	
**F25**	Heat shock protein HSP 90-alpha	gi|17865490		275	1	**R.ALLFVPR.R(20)**	**8%**
						**R.APFDLFENR.K(39)**	
						**R.RAPFDLFENR.K(41)**	
						**R.ELISNSSDALDK.I(89)**	
						**K.SLTNDWEDHLAVK.H(48)**	
**F27**	heat shock protein 70 [Liriomyza huidobrensis]	gi|89892741		225	3	**R.FEELCADLFR.S(68)**	**4%**
						**K.NQIHDIVLVGGSTR.I(68)**	
						**R.ARFEELCADLFR.S(58)**	
						**K.MDKNQIHDIVLVGGSTR.I + Oxidation (M)(31)**	
	Heat Shock Protein family member (hsp-1)	gi|17541098		219	2	**R.LSKDDIER.M(52)**	**6%**
	[Caenorhabditis elegans]					**R.FEELCADLFR.S(68)**	
						**R.ARFEELCADLFR.S(58)**	
						**K.SINPDEAVAYGAAVQAAILSGDK.S(41)**	
**F32**	DB:Trembl Frame:-1 orf:3 Homolog:Lipoprotein-		GH986605	19	1	**K.VLASIDLTGK.T(19)**	**9%**
	related protein Evalue:7e-07 Bitscore:56.2						
F35*	tropomyosin	gi|42559676		273	2	**K.IVELEEELR.V(55)**	**17%**
F36*, F53*						**K.LAMVEADLER.A(44)**	
						**R.EDSYEEQIR.L(74)**	
						**R.KLAMVEADLER.A(51)**	
						**K.ALQREDSYEEQIR.L(18)**	
	DB:Swissprot Frame:1 orf:2 Homolog:Tropomyosin-		GH986919	229	3	**R.IQLLEDDLER.T(69)**	**18%**
	1, isoforms 9A/A/B Evalue:3e-30 Bitscore:131					**K.LSEASQAADESER.A(93)**	
						**R.IQLLEDDLERTEER.L(67)**	
	DB:Swissprot Frame:2 orf:1 Homolog:Tropomyosin		GH986674	48	1	**R.LEDDLVHEK.E(35)**	**38%**
	Evalue:3e-11 Bitscore:66.6					**K.EVDRLEDDLVHEK.E(13)**	
**F36**	DB:Trembl Frame:2 orf:1 Homolog:Putative		EZ048810	109	2	**R.DYPIYNDIPSTR.F(51)**	**28%**
F33, F37, F64, F69, F71,	uncharacterized protein Evalue:1e-18 Bitscore:97.1					**K.QAGFYADAEAQCQVIR.R(58)**	
F72							
**F39**	DB:Trembl Frame:2 orf:3 Homolog:CG14304-PA		EZ048802	104	2	**K.DYPTYNEIPNTR.F(55)**	**32%**
F34, F42, E64, E90	Evalue:9e-18 Bitscore:95.5					**K.QAGFYADIDAQCQAIR.R(49)**	
**F42**	DB:Swissprot Frame:-2 orf:1 Homolog:Succinyl-CoA		GH986609	53	2	**K.EEQVQEAFR.I(35)**	**17%**
	ligase [GDP-forming] subunit beta, mitochondrial E					**K.LPIIAANSLEDAAAK.A(19)**	
**F51**	DB:Trembl Frame:2 orf:1 Homolog:AGAP009479-		EZ048819	58	1	**K.QAGFYADTDAQCQVIR.R(58)**	**16%**
E60, E63, E96, F31, F32,	PA Evalue:7e-15 Bitscore:83.2						
F52, F70, F73, F96							
**F54**	DB:Trembl Frame:1 orf:1 Homolog:Putative		EZ048790	62	1	**K.QAGFYADTEAQCQVIR.R(62)**	**18%**
F40, F41, F66, F67, F68	uncharacterized protein Evalue:2e-16 Bitscore:88.6						
**F55**	DB:Swissprot Frame:2 orf:1 Homolog:CD109		EZ048800	29	1	**R.TVVVYDYYNPQDR.K(29)**	**20%**
	antigen Evalue:2e-06 Bitscore:52.4						
**F56**	GTP-specific succinyl-CoA synthetase beta subunit	gi|4406564		59	1	**K.INFDDNAEFR.Q(59)**	**5%**
	[Homo sapiens]						
**F57**	heat shock protein 60 [Salmo salar]	gi|16923167		63	1	**K.VGGTSEVEVNEK.K(63)**	**7%**
F65*	14-3-3 protein beta/alpha-2 (Protein 14-3-3B2)	gi|82089139	(GH986681)	90	0	**R.NLLSVAYK.N(48)**	**6%**
	[Oncorhynchus mykiss]					**R.YDDMAGAMK.S(42)**	
**F73**	DB:Swissprot Frame:2 orf:2 Homolog:60S ribosomal		GH986676	21	1	**K.LSKDVSSSR.R(21)**	**6%**
	protein L26-1 Evalue:6e-45 Bitscore:179						
**F75**	DB:Swissprot Frame:2 orf:2		EZ048806	234	3	**R.LVEVPFLQDK.K(56)**	**33%**
	Homolog:Translationally-controlled tumor protein					**K.LVDNVLFEVTGK.Y(86) K.DAVTGDEMFSDSYK.Y(80)**	
	homolog Evalue:1e-57 Bi					**K.RVQEQSPNEVDQFK.T(8)**	
**F81**	DB:Swissprot Frame:2 orf:4 Homolog:Myosin		EZ048792	331	6	**K.DTFASLGR.A(30)**	**37%**
F80, F89	regulatory light polypeptide 9 Evalue:4e-50					**R.DLLGGVGDK.L(57)**	
	Bitscore:198					**K.GQLDYVAFAK.L(42)**	
						**K.LSADEMSQAFK.G + Oxidation (M)(67)**	
						**K.EAFTMMDQNR.D + Oxidation (M)(38)**	
						**K.VAGVDPEATITNAFK.L(97)**	
**F81**	DB:Swissprot Frame:2 orf:1 Homolog:Cathepsin L1		GH986678	98	1	**K.LPDLSEQNLVDCSK.K(98)**	**5%**
	Evalue:2e-65 Bitscore:249						
**F87**	DB:Swissprot Frame:3 orf:1 Homolog:Troponin C		GH986791	24	1	**R.QIGTLLR.T(24)**	**4%**
	Evalue:3e-60 Bitscore:231						
**F88**	putative LEA III protein isoform 2 [Corylus avellana]	gi|14148981		69	1	**K.AGESQVQDTANAAK.N(69)**	**16%**
	glycosyl transferase, family 2[Shewanella sediminis	gi|157373461		59	1	**R.HLLISLADK.Y(59)**	**1%**
	HAW-EB3						
	DB:Swissprot Frame:2 orf:5 Homolog:Myosin,						
	essential light chain Evalue:8e-30 Bitscore:131		EZ048813	404	10	**K.EVDEILR.L(20)**	**58%**
						**R.HLLLSLGEK.L(59)**	
						**K.ESNGTIIAAELR.H(68)**	
						**K.DVGTLEDFMEAMR.V(73)**	
						**K.LTVEEFMPIYGQLSK.E(28)**	
						**R.VFDKESNGTIIAAELR.H(26)**	
						**K.EKDVGTLEDFMEAMR.V(23)**	
						**K.EVFGVYDMFFGDGTNK.V(45)**	
						**K.KLTVEEFMPIYGQLSK.E(26)**	
						**K.EVFGVYDMFFGDGTNKVDAMK.V(37)**	
**F95**	SJCHGC06651 protein [Schistosoma japonicum]	gi|56759014		103	0	**K.NTTCEFTGDILR.T(52)**	**9%**
						**R.TVSGVNGPLVILDDVK.F(51)**	

Generated MS/MS data were searched against the NCBInr and tardigarde protein databases. Spot number, protein annotation, accession number, total protein score, number of matched peptides, peptide sequence and sequence coverage are listed. Identical proteins identified in different spots are listed only once and the spot with the highest protein score (in bold) is ranked at the top.

The 15 proteins which were identified in both databases are indicated with asterisk (e.g. spot A30*) and both accession numbers are listed. In these cases the listed peptide sequences belong to the hit with the highest score. Protein spots below the bold one are marked with °, when only found in the NCBInr database or marked with ∧, when only found in the tardigrade protein database.

Furthermore we were able to identify additional 150 protein spots by searching MS/MS data in the clustered EST database of *M. tardigradum*. These 150 proteins correspond to 36 unique contigs and ESTs. The protein information is listed in Table 3 and the protein spots are indicated by blue circles in the 2D reference map (Figure 5). Unfortunately, it was not possible to annotate them when performing a BLAST search. For these proteins of unknown function more information could be obtained by applying protein domain annotation methods. We ran all proteins through the DomainSweep pipeline which identifies the domain architecture within a protein sequence and therefore aids in finding correct functional assignments for uncharacterized protein sequences. It employs different database search methods to scan a number of protein/domain family databases. 2 out of the 36 unique proteins gave a significant hit, whereas 28 proteins were listed as putative and 6 proteins gave no hit at all.

**Table 3:** Identified proteins without annotation.

Spot no.	Accession no.	Total protein score	No. of unique/significant peptides	MS/MS peptide sequence (Indv. Ion score)	Sequence coverage	DomainSweep analysis
**A11**	GH986700	52	1	**-.VIAVSLPR.N(52)**	**3%**	**No hits**
A82, A88, B33, B41, B43, C50,						
D99, D105, E72, F87						
**A11**	GH986755	32	1	**-.LSISHNATLR.V(32)**	**4%**	**Putative**
						**IPR006210EGF**
**A94**	GH986643	39	1	**R.VDRSIPR.L(39)**	**3%**	**Putative**
A91, A95, A110, A123, A140,						**IPR004077 Interleukin-1 receptor, type II**
B49, B64, B83, B90, B98, B105,						
B155, B165, B173, B176, B185,						
B186, B187, B188, B189, B190,						
B191, B192, B193, B194, B195,						
C51, C128, C141, C153, D45,						
D46, D56, D57, D74, D123						
**A100**	EZ048767	229	4	**K.YDLIYK.G(15)**	**20%**	**Putative**
				**K.FLGFDTAGK.T(61)**		**IPR017956 AT hook, DNA-binding,**
				**K.IISFDVCNK.N(54)**		**conserved site**
				**K.TDSGVSCDVTDKCDPIVK.A(39)**		**IPR006689 ARF/SAR superfamily**
				**K.AVVDIEDPNNSAGDSIDYGK.Y(60)**		**IPR005464 Psychosine receptor**
**A112**	GH986667	317	5	**R.EQFTQGCTVGR.N(61)**	**22%**	**Putative**
A114				**K.LEAAPNQCPEYK.K(89)**		**IPR001749 GPCR, family 2, gastric**
				**K.KLEAAPNQCPEYK.K(64)**		**inhibitory polypeptide receptor**
				**K.IMEVCNEPNTYENVNR.F + Oxidation (M)(44)**		**IPR000372 Leucine-rich repeat, cysteine-**
				**K.IQSLCTPADLQFFQSTHDR.I(60)**		**rich flanking region, N-terminal**
						**IPR004825 Insulin/IGF/relaxin**
**A112**	EZ048821	98	2	**K.NADPLTILK.E(37)**	**14%**	**Putative**
				**K.IQSLCTPADLQFFQSTHDR.I(60)**		**IPR008355 Interferon-gamma receptor**
						**alpha subunit**
**A114**	EZ048817	49	1	**R.IGTETTSFDYLR.E(49)**	**3%**	**Putative**
						**IPR004354 Meiotic recombination protein**
						**rec114**
**A123**	EZ048785	221	4	**K.FLDFTR.G(28)**	**17%**	**Putative**
				**R.AADLDTLTK.L(57)**		**IPR000762 PTN/MK heparin-binding**
				**R.YLDMDQYDWDTR.S + Oxidation (M)(54)**		**protein**
				**R.GTFDTAHIQGLTALTTLR.L(60)**		
				**R.IMSVDLTDINSAPGMFDAAK.T + 2 Oxidation (M)(23)**		
**A136**	EZ048814	55	1	**R.IPAQFQSK.I(55)**	**5%**	**Putative**
						**IPR015874 4-disulphide core**
**B48**	EZ048766	273	5	**K.QVNAETFQK.A(36)**	**24%**	**Putative**
A157, A158, B49, B65				**K.YSETVHYEGGK.Q(39)**		**IPR000507 Adrenergic receptor, beta 1**
				**R.VDYVYSYHTK.M(4)**		**IPR000463 Cytosolic fatty-acid binding**
				**R.GDFWSTDKPHR.Y(32)**		**IPR004825 Insulin/IGF/relaxin**
				**K.YDIALDTVEATLK.S(70)**		
				**R.LIPDELLGTYEFSGK.Q(93)**		
**B61**	GH986621	231	6	**R.VLNNGVLR.V(39)**	**13%**	**Putative**
B60, B62, B64, B65, B79, B84,				**R.VITVPEGIK.V(49)**		**IPR001610 PAC motif (peptide matched in**
B93, B112, B143				**R.SLLGEIPITK.G(38)**		**frame 4)**
				**R.RVITVPEGIK.V(46)**		**IPR007758 Nucleoporin, Nsp1-like, C-**
				**R.VITVPEGIKVESFK.S(26)**		**terminal (peptide matched in frame 6)**
				**K.GSLTAGSSSNTSGSTGSSSYSSGTTGSSGTSGGK.T(34)**		
**B62**	EZ048776	230	6	**R.VLNNGVLR.V(39)**	**18%**	**Putative**
A138, B48, B60, B61, B64, B65,				**R.VITVPEGIK.V(49)**		**IPR007758 Nucleoporin, Nsp1-like, C-**
B84, B112, B138,B142, B143,				**R.SLLGEIPITK.G(38)**		**terminal**
B144, B161, B173				**R.RVITVPEGIK.V(46)**		
				**R.VEAPIQVDQLTADQQR.S(93)**		
				**R.VLNNGVLRVEAPIQVDQLTADQQR.S (69)**		
**B79**,	GH986933	38	1	**K.NGDVSIPR.Q(38)**	**6%**	**No hits**
D67, D109						
**B91**	GH986939	54	1	**R.EALSAVTGGR.R(62)**	**9%**	**No hits**
B43, B78-B80, B82, B83, B86,						
B87, B90, B92, B93, B97, B191,						
B193, C12, C51, C71, C112,						
C114, C123, C129, D2-D5, D8,						
D10, D21-D24, D27, D28, D31,						
D44, D47, D105, D118, D123,						
D124						
**B102**	EZ048815	403	6	**K.QVNAETFNK.A(40)**	**26%**	**Putative**
A23, A24, A26, A112, A127,				**K.GGPAWPKDEK.F(17)**		**IPR000507 Adrenergic receptor, beta 1**
B99, B103, B105, B107, B108,				**K.ILFRPTLSAR.A(36)**		**IPR006080 Mammalian defensin**
B110, B111, B144				**R.AQGLWEATTEGK.N(68)**		**IPR002181 Fibrinogen, alpha/beta/gamma**
				**R.LIPDELLGTFEFSGK.Q(92)**		**chain, C-terminal globular**
				**R.RLIPDELLGTFEFSGK.Q(36)**		**IPR000463 Cytosolic fatty-acid binding**
				**K.DYEFKEDGNMQMTAK.F + Oxidation (M)(20)**		
				**K.EVEYTSNYDMALDTVK.A(51)**		
				**R.MGLGVWESTSEQENMLEYLK.A(22)**		
				**R.GDKPGLAAFGDNIIEYSFTADSEGETGVLHGK.F(21)**		
**B103**	EZ048768	40	1	**R.VTTVSIPR.I(40)**	**3%**	**No hits**
B185, C150, C151, C153						
**B150**	GH986581	108	3	**R.VFVEEQLK.A(33)**	**14%**	**Putative**
B151, B173				**R.FNFLVFLGSTR.E(46)**		**IPR000990 Innexin**
				**R.GHTYEIMDPEK.V + Oxidation (M)(29)**		
**B152**	EZ048775	42	1	**R.KLEFILXFIF.-(42)**	**5%**	**Putative**
						**IPR003061 Colicin E1 (microcin) immunity**
						**Protein**
						**IPR000048 IQ calmodulin-binding region**
**B179**	GH986603	53	1	**R.AFEVPASECGK.S(53)**	**5%**	**Putative**
						**PR015880 Zinc finger, C2H2-like**
						**IPR008264 Beta-glucanase**
**B191**	EZ048789	26	1	**K.GSIGAPDVPK.N(26)**	**4%**	**Putative**
						**IPR001955 Pancreatic hormone**
**B186**	GH986708	468	6	**R.AFEVPASECGK.S(46)**	**25%**	**Putative**
A140				**R.AFEVPASECGKSPK.R(82)**		**IPR015880 Zinc finger, C2H2-like**
				**R.YRAFEVPASECGK.S(36)**		**IPR000436 Sushi/SCR/CCP**
				**K.IVSKDVCGSSPKPR.K(90)**		**IPR008264 Beta-glucanase**
				**R.SESGALWSEEQECTAK.F(62)**		**IPR000008 C2 calcium-dependent**
				**R.SESGALWSEEQECTAKFHPR.D(137)**		**membrane targeting**
				**R.VQVMDKDVGSSDDLVEQFECLTGPLVSSR.S+Oxidation (15)**		
**C18**	EZ048777	46	1	**R.NLADQAMSMGDGPLNFAK.A + 2 Oxidation (M)**	**8%**	**Putative**
						**IPR003569 Cytochrome c-type biogenesis**
						**Protein CcbS**
						**IPR002282 Platelet-activating factor**
						**receptor**
**C78**	GH986847	32	1	**K.SEVFPRIR.S(32)**	**3%**	**Putative**
B188, B173, C141						**IPR003916 NADH-ubiquinone**
						**oxidoreductase, chain 5**
**C86**	GH986916	196	4	**K.NPYLELTDPK.-(38)**	**12%**	**Putative**
				**K.TPEESEAPQAIR.R(68)**		**IPR000863 Sulfotransferase**
				**K.TPEESEAPQAIRR.K(58)**		**IPR003504 Glial cell line-derived**
				**K.VEKTPEESEAPQAIR.R(32)**		**neurotrophic factor receptor alpha 2**
**C95**	GH986921	35	1	**-.VIAVSLPR.N(30)**	**2%**	**No hits**
B18, B19, B47, B49, B138, C51,						
C62, C65, D107						
**C95**	GH986692	31	1	**K.TALITGASTGIGR.A(31)**	**5%**	**Significant**
						**IPR002347 Glucose/ribitol dehydrogenase**
						**IPR002198 Short-chain**
						**dehydrogenase/reductase SDR**
						**Putative**
						**IPR003560 2,3-dihydro-2,3-**
						**dihydroxybenzoate dehydrogenase**
						**IPR002225 3-beta hydroxysteroid**
						**dehydrogenase/isomerase**
**C110**	GH986711	31	1	**K.ERSPLANK.I(31)**	**4%**	**Putative**
						**IPR006210 EGF**
**C118**	EZ048824	45	0	**K.DSVAIGFPK.D(24)**	**7%**	**Putative**
				**K.ADEAGFTDAIK.A(21)**		**IPR003535 Intimin bacterial adhesion**
						**mediator protein**
**C141**	EZ048801	395	6	**R.NQVYQSMER.H(34)**	**22%**	**Putative**
C117, C145				**R.QNIDAIEIPR.L(78)**		**IPR002546 Myogenic basic muscle-**
				**K.DFLSAVVNSIQR.R(58)**		**specific protein**
				**R.LSQLAVDSVEIAK.D(74)**		**IPR000795 Protein synthesis factor, GTP-**
				**R.MTISEPFESAEALK.D + Oxidation (M)(72)**		**binding**
				**R.LEDVDDVLMSAFGMLK.A + 2 Oxidation (M)(26)**		
				**R.MTISEPFESAEALKDMIVR.L + 2 Oxidation (M)(15)**		
				**R.LQSSPTLSSLVDQDTFELIR.Q(37)**		
**C141**	GH986597	27	1	**-.TAVEAVVR.T(27)**	**4%**	**Putative**
						**IPR003065 Invasion protein B**
**C156**	EZ048804	277	5	**K.QFPFPISAK.H(43)**	**27%**	**Putative**
				**R.NELGAQYNFK.I(44)**		**IPR001610 PAC motif**
				**R.VIQAATEILPGK.-(73)**		**IPR001713 Proteinase inhibitor**
				**K.LGHFQQYDVR.L(60)**		**IPR000010 Proteinase inhibitor I25,**
				**K.DRNELGAQYNFK.I(52)**		**cystatin**
				**K.HTGGSDFLIADPEAQGVADAVR.S(4)**		**IPR001878 Zinc finger, CCHC-type**
**D87**	GH986563	35	1	**K.DNVPLFVGR.V(35)**	**4%**	**Putative**
						**IPR000215 Protease inhibitor I4, serpin**
**D110**	EZ048786	46	1	**R.FATPLILTGSK.D(3)**	**6%**	**Putative**
				**R.DVSPHPAACLTHSGR.V(43)**		**IPR002353 Type II antifreeze protein**
						**IPR002371 Flagellar hook-associated**
						**protein**
						**IPR000204 Orexin receptor**
**E9**	GH986691	257	7	**K.YANPQELR.Q(51)**	**31%**	**Putative**
D2-D5, D8, D18, D10, D13,				**K.SINVPQVEK.E(32)**		**IPR000980 SH2 motif**
D14, D15, D19-D23, D27, D28,				**K.QYWPYVDEKPR.M(46)**		**IPR000463 Cytosolic fatty-acid binding**
D31, D40, D47, E3, E4, E6, E7,				**K.KQYWPYVDEKPR.M(30)**		
E8, E10, E11, E12, E14, E15,				**R.DEDSFLYETPEAQNPIVQK.K(28)**		
E16, E18, E19, E60, E61, E63,				**K.RDEDSFLYETPEAQNPIVQK.K(37)**		
E64, F31, F94, F95				**K.GLESETEDTAATTILIADMVHYLK.Y(33)**		
**F6**,	GH986624	35	1	**R.ESLDFFR.V(35)**	**3%**	**No hits**
F48						
**F63**	GH986878	38	1	**K.AEETVPVLLTAEEK.L(38)**	**7%**	**Significant**
						**IPR007327 Tumor protein D52**
						**Putative**
						**IPR004077 Interleukin-1 receptor, type II**

Generated MS/MS data were searched against the tardigrade clustered database. Spot number, protein annotation, accession number, total protein score, number of matched peptides, peptide sequence and sequence coverage are listed. Identical proteins identified in different spots are listed only once and the spot with the highest protein score (in bold) is ranked at the top. The significant or putative candidates found in Domain Sweep are also listed in the Table.

In addition, we analyzed further 185 protein spots, which are indicated with red colour in Figure 5. Despite high quality MS/MS spectra, it was not possible to identify these protein spots in either of the databases used in our study.

In summary, we identified 421 (69.5%) out of 606 protein spots which were picked from the preparative 2D gel. 271 spots yielded 144 unique proteins with distinct functions whereas 150 spots were identified as proteins with yet unknown functions.

### Functional Assignment of Proteins

**Figure 7:**
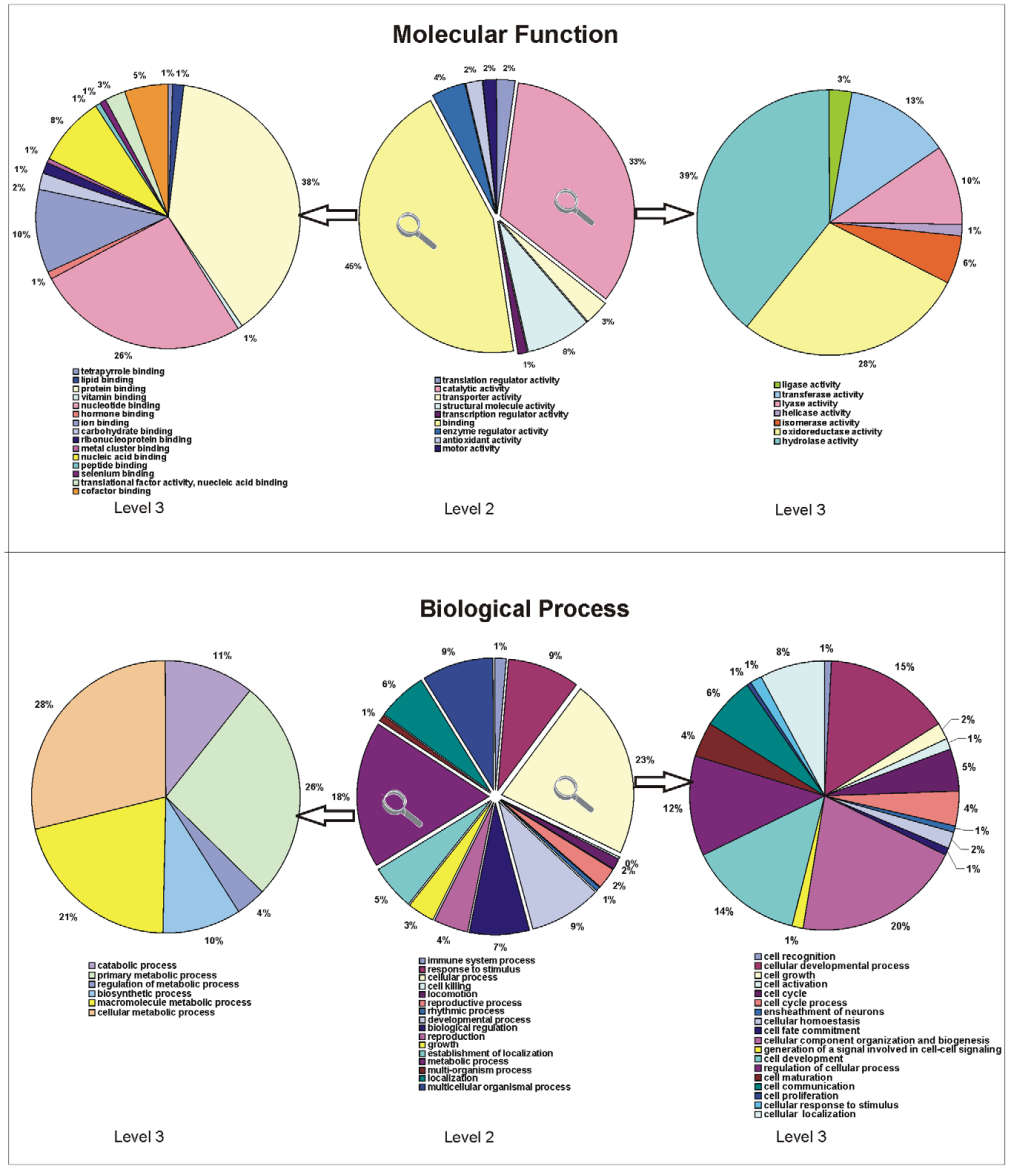
GO analysis of proteins identified in *M. tardigradum*. A total of 271 spots representing 144 unique proteins was analysed with the Blast2GO program. The GO categories “molecular function” and “biological process” are shown as pie charts. A total of 9 different molecular function groups and 16 groups for biological processes are present in our result. The major parts of these categories (level 2) are shown in more detail (level 3) on the left and right side.

The 144 unique proteins with annotation were further analysed using the Blast2GO program, which provides analysis of sequences and annotation of each protein with GO number to categorize the proteins in molecular function, biological process and cellular component. By analysing the proteins on the GO level 2 in the category molecular function we received a total of 9 subgroups as shown in Figure 7, upper middle chart. The majority of the identified proteins exhibit either binding (45%) or catalytic activity (33%). A more detailed analysis (GO level 3) revealed that 39% of the proteins with catalytic activity are involved in hydrolase activity (Figure 7, upper right chart) and 38% of binding proteins bind to other proteins (Figure 7, upper left chart).

Identified proteins are involved in diverse biological processes. A total of 16 subgroups of biological processes are represented (Figure 7, lower middle chart). 23% are involved in cellular processes and 18% in metabolic processes. Within the cellular processes a majority of 20% of tardigrade proteins are involved in cellular component organization and biogenesis. Within the metabolic processes 28% of proteins are involved in cellular metabolic processes, 26% in primary metabolic processes and 21% in macromolecule metabolic processes (Figure 7, lower right chart). A detailed GO description of all identified and annotated tardigrade proteins is included in Table S1.

### Identified Proteins and Protein Families

In our proteomic study several heat shock proteins have been identified, namely hsp-1 (spot F27), hsp-3 (spot F21), hsp60 (spot F57), hsp70 (spot B146, B173, C131, C133), hsp82 (spot F13), hsp86 (spot F24, F25), hsp90 alpha (spot E64), hsp90 beta (spot F24) and hsp108 (spot F12). Hsp70 is already described in *M. tardigradum* as a molecular chaperone which could play a role in desiccation tolerance [18]. Hsp60 could be identified in spot F57 when searching the corresponding MS/MS data against the NCBInr database. No hit was obtained in the tardigrade EST or protein database which is surprising, because hsp60 is an abundant protein.

Several protein spots have been identified as cytoskeletal proteins, including actin as most abundant protein spot (E48) on the 2D gel and tubulin. Actin and tubulin are highly conserved proteins and were used to control proteolytic degradation during our workup procedure by Western blotting. Four different actin proteins are found by MS/MS analysis, which play important roles in muscle contraction, cell motility, cytoskeletal structure and cell division. Tubulin is a key component of the cytoskeletal microtubules. Both alpha- and beta-tubulin could be identified on the 2D gel in spot D107, D110 and F6. Further proteins involved in motor activity and muscle contraction were found, namely tropomyosin (e.g. spot F35), myosin (e.g. spot F81), annexin A6 (e.g. spot D90) and myophilin (e.g. spot A128), which is a smooth-muscle protein and was described in the tapeworm *Echinococcus granulosus* [20].

In addition, several proteins have been identified which are known to have important roles in embryonic or larval development. Mitochondrial malate dehydrogenase precursor (e.g. spot B109), vitellogenin 1 and 2 (e.g. spot D62 and B88), GDP-mannose dehydratase (spot C87), protein disulfide isomerase 2 (e.g. spot F3), hsp-3 (spot F21), hsp-1 (spot F27), tropomyosin (spot F35) and troponin C (spot F87) belong to this group of proteins. Vitellogenin, a major lipoprotein in many oviparous animals, is known as the precursor of major yolk protein vitellin [21]. Vitellogenin is a phospholipo-glycoprotein which functions as a nutritional source for the development of embryos [22]. During developing oocytes vitellogenin and vitellin are modified through cleavage and by different posttranslational modifications (PTMs) like glycosylation, lipidation and phosphorylation. Interestingly we could identify vitellogenin in several spots on the 2D gel showing vertical (pI) shifts most probably caused by PTMs.

Peroxiredoxins identified first in yeast [23] are conserved, abundant, thioredoxin peroxidase enzymes containing one or two conserved cysteine residues that protect lipids, enzymes, and DNA against reactive oxygen species. Different isoforms of peroxiredoxins could be identified on the 2D gel: peroxiredoxin-4 (spot C132), peroxiredoxin-5 (spot B183) and peroxiredoxin-6 (spot D159). An important aspect of desiccation tolerance is protection against free radicals [24], [25]. Notably, the expression of 1-cysteine (1-Cys) peroxiredoxin family of antioxidants is reported in *Arabidopsis thaliana* and is shown to be related to dormancy [26]. Our results show the presence of important antioxidant systems, including superoxide dismutase (SOD) and peroxidases. Additionally different forms of glutathione S-transferases (spot A122, B153, B166, B169, D166, and D159) could be identified. Glutathione transferases (GSTs) constitute a superfamily of detoxifying enzymes involved in phase II metabolism. Detoxification occurs by either glutathione conjugation, peroxidase activity or passive binding [27]. Furthermore GSTs have cellular physiology roles such as regulators of cellular pathways of stress response and housekeeping roles in the binding and transport of specific ligands [28]. The consequence of this diversity in role is the expression of multiple forms of GST in an organism. It has been shown that the expression of the different isoenzymes is highly tissue-specific [29], and this heterogeneity of GSTs may be further complicated by posttranslational modifications such as glycosylation [30].

Some protein spots were identified as calreticulin (e.g. spot F14) which is a Ca^2+^-binding protein and molecular chaperone. Calreticulin is also involved in the folding of synthesized proteins and glycoproteins [31].

Three different cathepsin proteins could be identified: cathepsin K (spot A84), cathepsin Z (spot E80) and cathepsin L1 (spot F81). Cathepsin L is a ubiquitous cysteine protease in eukaryotes and has been reported as an essential protein for development in *Xenopus laevis* [32], *Caenorhabditis elegans* [33] and *Artemia franciscana* [34].

Several protein spots are associated with ATP generation and consumption and may have important roles in the early development as described for *Artemia*, because many important metabolic processes require ATP [35], [36]. ATP synthase (spot B152) regenerates ATP from ADP and Pi [37]. It consists of two parts: a hydrophobic membrane-bound part (CF0) and a soluble part (CF1) which consists of five different subunits, alpha, beta (spot E89), gamma, delta (spot C139) and epsilon. Arginine kinase (spot B167) is an ATP/guanidine phosphotransferase that provides ATP by catalyzing the conversion of ADP and phosphorylarginine to ATP and arginine [38]. The presence of arginine kinase has been shown in tissues with high energy demand [39].

Interestingly, we could identify the translationally controlled tumor protein (TCTP) (spot F75) on the 2D gel. TCTP is an important component of TOR (target of rapamycin) signalling pathway, which is the major regulator of cell growth in animals and fungi [40].

### Evaluation of Heat Shock Proteins by Western Blot Analysis

**Figure 8:**
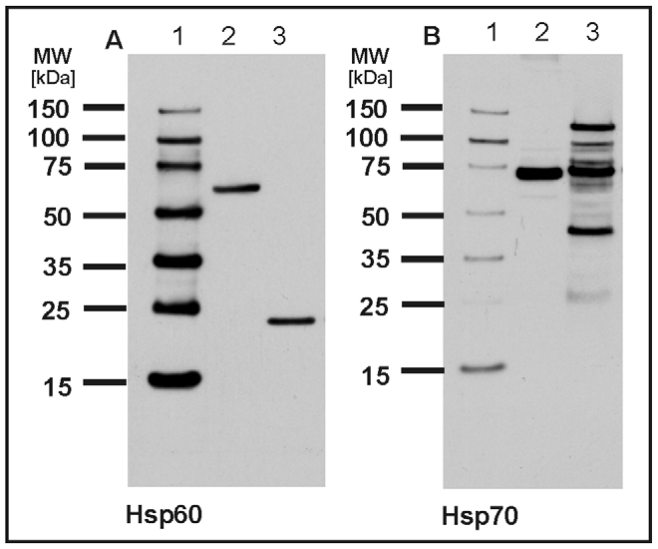
Detection of hsp60 and hsp70 by Western blotting. Total protein extract of *M. tardigradum* in the active state was separated on a one-dimensional polyacrylamide gel. Hsp60 (A) and hsp70 (B) could be immunodetected with high sensitivity. Lane 1A and 1B: DualVue Western blotting marker. Lane 2A and 2B: Total protein extract of HeLa cells. Lane 3A and 3B: Total protein extract of tardigrades. Notably, the protein bands in the HeLa control lysate show molecular weights of 60 and 70 kDa as expected. In contrast the detected protein band for hsp60 in *M. tardigradum* is considerably smaller. For hsp70 multiple bands are observed in *M. tardigradum* at higher as well as at lower molecular weights.

To evaluate the highly conserved heat shock proteins 60 and 70, we performed Western blot analyses with antisera directed against these proteins. Hsp70 was found in several spots on the reference 2D proteome map, e.g. in spot B172, C31, C133 and F27. None of these spots fits well to the calculated molecular weight of approx. 70 kDa, most of them were considerably smaller. In contrast, the immunoblot shows the strongest band at the expected position which is in agreement with the position of hsp70 in the control lysate of HeLa cells (Figure 8B). However, several additional bands can be observed at higher as well as at lower molecular weights. The lower bands might account for the identified spots on the 2D gel with lower molecular weight. The full-length protein might have escaped the spot picking procedure since only a limited number of detected spots were further processed.

**Figure 9:**
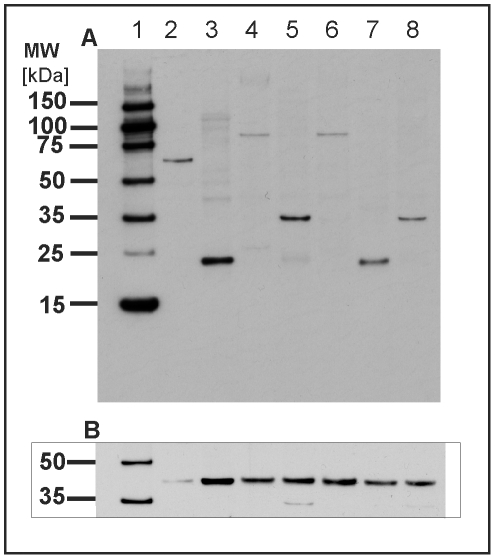
Detection of hsp60 in six different tardigrade species by Western blotting. Total protein extracts of tardigrades in the active state were separated on a one-dimensional polyacrylamide gel. Hsp60 (A) and actin (B) as loading control were immunodetected with high sensitivity. Lane 1: DualVue Western blotting marker. Lane 2: Total protein extract of HeLa cells. Lane 3: Total protein extract of *M. tardigradum*. Lane 4: Total protein extract of *Paramacrobiotus richtersi*. Lane 5: Total protein extract of *Paramacrobiotus* “*richtersi* group” 3. Lane 6: Total protein extract of *Macrobiotus tonollii*. Lane 7: Total protein extract of *Paramacrobiotus* “*richtersi* group” 2. Lane 8: Total protein extract of *Paramacrobiotus* “*richtersi* group” 1. Interestingly, the detected protein bands were ranging from 100 kDa to less than 24 kDa. Only hsp60 in the HeLa control lysate was detected at its expected position at 60 kDa.

Hsp60 was identified in spot F57 of the 2D map as described above. Since hsp60 was identified by only one peptide hit we confirmed this result by immunostaining using an antibody directed against a peptide in the C-terminal region of the entire protein. Only one band is visible on the Western blot at approx. 24 kDa whereas the protein band in the HeLa control lysate is located at its expected position (Figure 8A). The lower molecular weight is in accordance with the location of hsp60 (spot F57) on the 2D gel. Thus, in *M. tardigradum* hsp60 exists in a significantly shorter form. Whether the observed difference in the molecular weight indicates a different function and role of this protein in *M. tardigradum* needs to be investigated in future experiments. To test whether other tardigrade species show similar results we performed an immunoblot with protein lysates from 5 other species namely *Paramacrobiotus richtersi*, *Paramacrobiotus* “*richtersi* group” 3, *Macrobiotus tonollii*, *Paramacrobiotus* “*richtersi* group” 2 and *Paramacrobiotus* “*richtersi* group” 1. Total protein lysate from HeLa cells was loaded as control (Figure 9A, lane 1). Actin served as loading control for all lysates (Figure 9B). Interestingly, some species also exhibit truncated forms of hsp60 on the Western blot whereas others show higher forms. The molecular weights are ranging from approx. 75 kDa for *P.* “*richtersi* group” 2 and *P.* “*richtersi* group” 1 lysates (Figure 9A, lane 4 and 6), 35 kDa for *P.* “*richtersi* group” 3 and *P. richters*i lysates (Figure 9A, lane 5 and 8) down to 24 kDa for *M. tardigradum* and *M. tonollii* (Figure 9, lane 3 and 7).

## Discussion

### Establishing a Comprehensive Proteome Map of *Milnesium tardigradum*

The analysis of the proteome *of M. tardigradum* represents to our knowledge the first detailed study of tardigrades on the protein level. Our experimental strategy aimed to identify as many as possible proteins from tardigrades. Thus, we have not employed any subcellular fractionation steps to obtain specific subproteomes. We have tested various protocols for protein extraction from whole tardigrades. We could show that direct homogenisation of tardigrades in lysis buffer without any previous precipitation steps is most efficient and enables the generation of high quality 2D gels. Since nothing was known about the proteolytic activity in *M. tardigradum* special precautions were taken to avoid any protein degradation or proteolysis throughout the whole workup procedure. Integrity of proteins was carefully inspected by Western blot analysis of the two housekeeping proteins actin and tubulin where the sequence homology was assumed to be high enough to detect the proteins with commercially available antibodies. The development of a robust workup protocol laid the basis for the generation of a protein map from whole tardigrades in the active state. 56 unique proteins could be identified by searching high quality MS/MS spectra against the publicly available NCBInr database. However, for many proteins we could not find any homologues in the NCBInr database and only by using our own newly generated tardigrade protein database it was possible to identify another 73 unique proteins. 15 proteins were present in both databases. In addition 36 unique proteins were found in the clustered tardigrade EST database which could not be annotated by BLAST search. This concerns new specific proteins of *M. tardigradum*.

### Performance of Database Searches

When we started our study of the tardigrade proteome very little was known about tardigrades at the genome and gene expression level. To this day, only 12 proteins are recorded in the NCBInr database, which originate from *M. tardigradum*. For all of them only partial sequences ranging from as few as 43 amino acids for beta actin up to 703 amino acids for elongation factor-2 are available. Therefore, in parallel to our proteomic study a *M. tardigradum* EST sequencing project has been initiated. Subsequently, two tardigrade specific databases have been established: a clustered tardigrade EST database and a tardigrade protein database which was extracted from the clustered EST database and thus represents a subdatabase containing all tardigrade-specific proteins with annotated function. However, since cDNA sequencing is still ongoing sequence information remains incomplete. We assume that the tardigrade database currently covers approximately one tenth of the tardigrade specific genes comparing the unique clusters found in tardigrades to all known proteins of *Caenorhabditis elegans* or *Drosophila melanogaster* in Ensembl. This fact is greatly influencing our database searches. For most of the protein spots that were analysed by ESI-MS/MS high quality fragmentation spectra were obtained from MS/MS experiments. However, when we searched these MS/MS data against the tardigrade databases and the publicly available NCBInr database, only about 70% of the spots yielded in protein identification whereas the remaining spots gave no significant protein hit. In addition it was impossible to manually extract peptide sequences that were sufficient in length to perform BLAST searches with satisfactory results.

**Figure 10:**
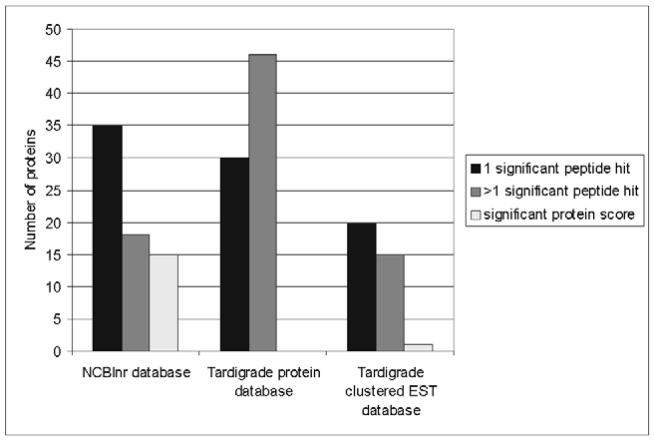
Statistical analysis of significant peptides found in the three different databases which were used to search the MS/MS data. The number of significant peptide hits is compared between the different databases. When searching against the NCBInr database most proteins were identified with only one significant peptide hit. In contrast when using the tardigrade protein database most proteins were represented by two or more significant peptides.

When we examined the protein hits obtained by the three databases in more detail we found that in the NCBInr database approximately one half of the proteins were identified by only one significant peptide hit (Figure 10). For about 25% of the proteins more than one significant peptide hit was obtained. For the remaining 25% only the protein score which is the sum of two or more individual peptides scores was above the significance threshold while none of the peptide scores alone reached this value. In contrast, proteins found in the tardigrade protein database were predominantly identified by more than one significant peptide hit whereas a smaller number was represented by only one peptide. In no cases a protein was identified by the sum of non-significant peptide matches. For proteins without annotation the number of proteins identified by only one peptide was only slightly higher than the number of proteins identified by two or more peptides.

These results are not surprising. Since the NCBInr database contains very few sequences originating from *M. tardigradum* e.g. elongation factor 1-alpha the identification relies predominantly on high homologies between tardigrade sequences and sequences from other more or less related species of other taxa. The chances for detecting more than one identical peptide is significantly higher when searching MS/MS data against the tardigrade EST and tardigrade protein databases since these databases contain only tardigrade specific sequences.

Overall, one might evoke a potentially high false positive rate especially since proteins are included in the reference map which are either identified by only one significant peptide hit or where two or more non-significant peptide scores are summed up to a significant protein score. On the other hand, proteins like LEA and heat shock protein 60 are identified by only one peptide match. Nevertheless they could be confirmed by Western blot analysis to be present in the tardigrade protein extract. Given the incomplete sequence data available to date many proteins might escape confirmation by orthogonal methods e.g. due to the lack of specific antibodies.

### Proteins Associated with Anhydrobiosis

Among the numerous proteins which were identified in our proteomic study some proteins have already been reported to play an important role in anhydrobiotic organisms. Most importantly, spot F88 was identified as a protein belonging to the LEA (late embryogenesis abundant) family (group 3). This result was already known from Western blot analyses (Schill et al., 2005, poster presentation, ISEPEP, Denmark). At least six different groups of LEA proteins have been described so far. Group 1, 2 and 3 are the three major groups. Whereas group 1 is only found in plants and group 2 predominantly in plants, group 3 is reported in organisms other than plants. Although the precise role of LEA proteins has not yet been fully elucidated, different research groups have reported on their association with tolerance to water stress by desiccation [41], [42]. LEA protein of group 3 could be already identified in nematodes *C. elegans*, *Steinernema feltiae* and *Aphelenchus avenae*, and the prokaryotes *Deinococcus radiodurans*, *Bacillus subtilis* and *Haemophilus influenzae* [43], [44], [45].

### Proteins Exhibiting an Unusual Location on the 2D Map

In general we identified some proteins which show a lower molecular weight than expected. As described above hsp60 is detected as a protein band at 24 kDa by Western blotting and the location of the corresponding spot on the 2D gel shows the same molecular weight. Comparison of different tardigrade species indicates the existence of short as well as long forms of hsp60.

Unique proteins, when analyzed on the 2D gel, often show multiple spots due to posttranslational modifications. Proteins of the vitellogenin family are widely distributed on the 2D gel and show pI as well as molecular weight shifts, which are due to modification through cleavage and to different PTMs like glycosylation and phosphorylation during development of oocytes. Ongoing experiments to detect PTMs using different fluorescence staining methods like ProQ-Emerald for the detection of glycoproteins and ProQ-Diamond for the detection of phosphoproteins indicate that these modifications indeed occur in tardigrades (data not shown).

### Prediction of Functional Domains in Proteins with Yet Unknown Functions

36 proteins which could not be identified by BLAST searches were further examined looking for matching functional protein domains with DomainSweep. The function of the following two spots could be revealed with high confidence (Table 3): spot F63 seems to belong to the “tumor protein D52” interpro family (IPR007327). The hD52 gene was originally identified through its elevated expression level in human breast carcinoma, but cloning of D52 homologues from other species has indicated that D52 may play roles in calcium-mediated signal transduction and cell proliferation. Regarding the taxonomic neighbours of the tardigrades, one member in *C. elegans* and 10 members in *Drosophila melanogaster* are reported by Interpro for this family. Spot C95 seems to belong to the family “glucose/ribitol dehydrogenase” (IPR002347). 80 members both in *C. elegans* and in *Drosophila melanogaster* are reported for this family. 28 putative hits were found associated with other spots. These protein hits are putative candidates and therefore less reliable. A comprehensive protein database of *M. tardigradum* as the result of our ongoing cDNA sequencing will help us to evaluate these candidates.

### Conclusion

In this study we present for the first time a comprehensive proteome map of *M. tardigradum*. A full description of proteins present in the active state provides a valuable basis for future studies. Most importantly, the protein reference map allows us to undertake quantitative proteomics analysis to detecting proteins with different expression levels in the active versus the anhydrobiotic state. In particular, our workflow is fully compatible with the application of 2D difference gel electrophoresis (2D DIGE), which is one technique allowing sensitive analysis of differences in the protein expression levels. This differential analysis on the protein level will help us to understand survival mechanisms in anhydrobiotic organisms and eventually to develop new methods for preservation of biological materials.

## Materials and Methods

### Tardigrade Culture and Sampling

Tardigardes of the species *M. tardigradum* Doyère 1840 were maintained in a laboratory culture. The culture was grown on agarose plates (3%) (peqGOLD Universal Agarose, peqLAB, Erlangen Germany) covered with Volvic™ water (Danone Waters, Wiesbaden, Germany) at 20°C. The juveniles were fed on green algae *Chlorogonium elongatum*, the adults with bdelloid rotifers *Philodina citrina*. The specimens for the experiments were all of middle-age, thus effects of age can be excluded. Tardigrades were starved over 3 days and washed several times with Volvic™ water to avoid contamination with food-organisms. Subsequently the animals were transferred to microliter tubes (200 individuals per tube) and surrounding water was reduced to approx. 1–2 µl. An active state (I) according to Schill et al. [18] was investigated in this work. All samples were shock frozen in liquid nitrogen and stored at −80°C. 200 individuals are defined as one aliquot. Other tardigrade species (*Paramacrobiotus richtersi*, *Paramacrobiotus* “*richtersi* group” 3, *Macrobiotus tonollii*, *Paramacrobiotus* “*richtersi* group” 2 and *Paramacrobiotus* “*richtersi* group” 1) used for immunodetection of hsp60 were prepared in the same way.

### Sample Preparation for Gel Electrophoresis

To optimize the sample preparation different precipitation methods have been tested. Chloroform/methanol and TCA/acetone precipitations were performed as described by Wessel, Fluegge [46] and Görg [47], respectively. We used also the commercially available precipitation kit (clean-up kit from GE Healthcare). Comparing the result of different precipitation protocols on a 1D gel we decided to homogenise the tardigrades directly in ice cold lysis buffer and avoid any precipitation steps. The animals (200 individuals) were homogenised directly in 60 µl lysis buffer (containing 8 M urea, 4% CHAPS, 30 mM Tris, pH 8,5) by ultrasonication (SONOPULS, HD3100, Bandelin Electronic) with 45% amplitude intensity and 1–0.5 sec intervals. The lysis buffer contained a Protease Inhibitor Mix (GE Healthcare) to inhibit serine, cysteine and calpain proteases. After homogenisation the samples were stored at −80°C. For gel electrophoresis insoluble particles were removed by centrifugation for 2 min at 14,000 g and the supernatant was quantified using BCA mini-assay.

### One Dimensional Gel Electrophoresis and Western Blotting

To compare the efficiency of different sample preparation methods we separated approx. 10 µg total protein extract on a 1D gel. The gel was stained with protein staining solution (PageBlue from Fermentas). For Western blotting a total protein extract of tardigrades (15–20 ug) was separated on a NuPAGE™ 4–12% Bis-Tris mini gel (Invitrogen) using MES running buffer. 200 V were applied until the bromophenol blue front had reached the bottom of the gel (approx. 40 min). Separated proteins were electro transferred onto PVDF membrane for 1.5 h at maximum 50 mA (0.8/cm^2^) in a semi-dry transfer unit (Hoefer™ TE 77) using following transfer solution: 24 mM Tris, 192 mM glycine and 10% methanol. The PVDF membrane was incubated in a blocking buffer containing 5% non-fat milk, 0.1% Tween20 in PBS. As primary antibodies we used anti actin pan Ab-5 (dianova), anti hsp 60 Ab (D307) (Cell signaling), anti hsp70 Ab (BD Biosciences Pharmingen) and anti α-Tubulin Ab (Sigma).

For molecular weight determination of the target proteins on film we used ECL DualVue marker (GE-Healthcare). Immunoreaction was detected using the ECL Western Blotting Detection kit from GE Healthcare. Images were acquired using an Image Scanner Model UTA-1100 (Amersham Biosciences).

### Two Dimensional Gel Electrophoresis

For 2D gel preparation we added 60 µl 2x sample buffer (7 M urea, 2 M thiourea, 2% CHAPS, 2% DTT, 2% IPG-buffer 3–11 NL) to each aliquot and incubated by shaking for 30 min at 25°C. To avoid streaking on the gels we used 330 µl destreaking buffer (GE Healthcare) instead of rehydration buffer, to which we added 2% IPG-buffer (pI 3–11). Samples were incubated by shaking for 30 min at 25°C. We loaded 100 µg protein on analytical gels and 330 µg on preparative gel.

#### Strip loading

Loading of proteins was performed during strip rehydration with the recommended volume (450 µl for 24 cm strips) over night.

#### IEF conditions

First dimension isoelectric focusing (IEF) was performed, using 24 cm long IPG strips with non-linear gradients from pH 3–11 and an Ettan IPGphor instrument and proceeded for 46.4 kVh with the following running protocol: 3 h at 300 V, 6 h at 500 V, 8-h gradient up to 1000 V, 3-h gradient up to 8000 V and 3 h at 8000 V. Strips were either immediately used for the second dimension or stored at −80°C.

#### Second dimension

Strips were equilibrated in 6 M urea, 2% SDS, 30% glycerol, 0.375 M Tris-HCl pH 8.8, 0.002% bromophenol blue and 10 mg/ml DTT for 15 min, followed by a second equilibration step with the same buffer containing 25 mg/ml iodoacetamide instead of DTT, also for 15 min.

Strips were loaded on 12% SDS-gels with an overlay of agarose solution (0,5 mg/100 ml electrophoresis buffer). The second dimension was performed using an Ettan Dalttwelve electrophoresis system (GE Healthcare). Separation was carried out at 1.5 watt/1.5 mm thick gel until the bromophenol blue reached the bottom of the gel (approx. 18 h).

#### Silver staining of proteins and image analysis

Proteins on analytical gels were visualized by destructive silver staining according to Blum [48]. Additionally, we performed a silver stain compatible with mass spectrometric analysis described by Sinha [49] for preparative gels. Images were acquired using an Image Scanner Model UTA-1100 (Amersham Biosciences).

### Protein Identification

#### In-gel digestion

Protein spots were excised semi-manually with a spot picker (GelPal, Genetix) following non-destructive silver staining and stored at −80°C after removing water. Gel pieces were reduced, alkylated and in-gel digested with trypsin. Briefly, after incubation with 150 µl water at 42°C for 8 min, water was removed (washing step) and gel pieces were shrunk by dehydration with 150 µl 40 mM NH_4_HCO_3_/ethanol 50∶50 (v/v) at 42°C for 5 min in a thermo mixer (600 rpm). The solution was removed and the proteins were reduced with 50 µl 10 mM dithiothreitol in 40 mM NH_4_HCO_3_ for 1 h at 56°C. The solution was removed and gel pieces were incubated with 150 µl 40 mM NH_4_HCO_3_ for 5 min at 42°C. After removing the solution gel pieces were alkylated with 100 µl 55 mM iodoacetamide in 40 mM NH_4_HCO_3_ for 30 min at 25°C in the dark, followed by three alternating washing steps each with 150 µl of 40 mM NH_4_HCO_3_ and ethanol for 5 min at 37°C. Gel pieces were then dehydrated with 100 µl neat acetonitrile for 1 min at room temperature, dried for 15 min and subsequently rehydrated with porcine trypsin (sequencing grade, Promega, Mannheim, Germany) with the minimal volume sufficient to cover the gel pieces after rehydration (100 ng trypsin in 40 mM NH_4_HCO_3_). Samples were incubated over night at 37°C.

#### Extraction

After digestion over night the supernatant was collected in PCR-tubes while gel pieces were subjected to four further extraction steps. Gel pieces were sonicated for 5 min in acetonitrile/0.1% TFA 50∶50 (v/v). After centrifugation the supernatant was collected and gel pieces were sonicated for 5 min in acetonitrile. After collecting the supernatant gel pieces were sonicated for 5 min in 0.1% TFA followed by an extraction step again with acetonitrile. The combined solutions were dried in a speed-vac at 37°C for 2 h. Peptides were redissolved in 6 µl 0.1% TFA by sonication for 5 min and applied for ESI-MS/MS analysis.

#### ESI-MS/MS analysis and database search

NanoLC-ESI-MS/MS was performed on a Qtof Ultima mass spectrometer (Waters) coupled on-line to a nanoLC system (CapLC, Waters). For each measurement 5 µl of the digested sample was injected. Peptides were trapped on a Trapping guard C18- AQ, 10 mm×0.3 mm, particle size 5 µm (Dr. Maisch). The liquid chromatography separation was performed at a flow rate of 200 nl/min on a Reprosil C18-AQ column, 150 mm×75 µm, particle size 3 µm (Dr. Maisch GmbH). The following linear gradient was applied: 5% B for 5 min, from 5 to 15% B in 5 min, from 15 to 40% B in 25 min, from 40 to 60% B in 15 min and finally 60 to 95% B in 5 min. Solvent A contains 94.9% water, 5% acetonitrile, 0.1% formic acid, solvent B contains 95% acetonitrile, 4.9% water and 0.1% µl formic acid. The LC-ESI-MS/MS device was adjusted with a PicoTip Emitter (New Objective, Woburn, MA) fitted on a Z-spray nanoESI interface (Waters). Spectra were collected in the positive ion mode. The capillary voltage was set to 2400 V and the cone voltage was set to 80 V. Data acquisition was controlled by MassLynxTM 4.0 software (Waters). Low-energy collision-induced dissociation (CID) was performed using argon as a collision gas (pressure in the collision cell was set to 5×10^−5^ mbar), and the collision energy was in the range of 25–40 eV and optimized for all precursor ions dependent on their charge state and molecular weight. Mass Lynx raw data files were processed with Protein Lynx Global Server 2.2 software (Waters). Deisotoping was performed using the MaxEnt3 algorithm.

The obtained MS/MS spectra were searched against the publicly available NCBInr database using the MASCOT algorithm version 2.0 (Matrix Science, London, UK). The mass tolerance was set to 0.1 Da for fragment ions and 200 ppm for precursor ions. No fragment ions score cutoff was applied. The following search parameters were selected: variable modification due to methionine oxidation, fixed cysteine modification with the carbamidomethyl-side chain, one missed cleavage site in the case of incomplete trypsin hydrolysis. The following settings were applied: minimum protein score >53, minimum number of peptides ≥1. Furthermore, protein hits were taken as identified if a minimum of one peptide had an individual ion score exceeding the MASCOT identity threshold. Under the applied search parameters a sum MASCOT score of >53 refers to a match probability of p<0.05, where p is the probability that the observed match is a random event. Redundancy of proteins that appeared in the database under different names and accession numbers was eliminated. Additionally we searched against the *M. tardigradum* EST and protein database (see below) to identify sequences not present in the NCBInr databases. The following settings were applied: minimum protein score >14 for the EST and >27 for the clustered EST database (p<0.05). Other parameters were as described for the NCBInr searches.

### Generation of the Tardigrade EST Database

cDNA libraries from mRNA from tardigrades in different states (active, inactive, transition states) were prepared and sequenced (Mali et al, submitted data). The obtained EST sequences were cleaned from vector sequences using Seqclean against UniVec-database from NCBI (version 12. September 2008, Kitts et al., unpublished). Repeats within the cleaned ESTs were masked using the online service RepeatMasker (version 3.2.6, RM-20080801, Smit et al., unpublished data) followed by a second Seqclean run to eliminate low quality and short sequences. The assembly was performed using cap3 [50] with clipping enabled and resulted in 3318 Unigenes (2500 singlets, 818 contigs). Identification of ribosomal sequences was done using a BlastN-search [51] against the Silva-DB (only eukaryotic sequences, Silva95, [52]) and an E-value cutoff of 1e-3 and resulted in 46 sequences which showed high similarity to ribosomal sequences. Unigenes coding for known proteins were identified using a BlastX search against Uniprot/Swissprot (version 14.1, September 2008), Uniprot/TrEMBL (version 56.1, September 2008, The UniProt Consortium, 2008) and NRDB (version 12. September 2008,) with an E-value cutoff of 1e-3 and a hmmer-search against PFAM database (release 22, [53]) with an E-value cutoff of 0.1. Translation of Unigen sequences which gave a BlastX or PFAM hit (1539/1889 sequences) into the corresponding frame and a six-frame translation was performed using Virtual Ribosome (version 1.1 Feb-Mar, 2006, [54]). For six frame translation the read through mode of Virtual Ribosome was used. Afterwards stop codons were substituted by an undefined amino acid (X). All new sequences have been deposited in GenBank. The accession numbers are indicated in the Tables 2, 3 and S1 in the column “Tardigrade specific Accession no.”.

### Classification of Proteins

For functional analysis of identified proteins we used Blast2GO software, which consists of three main steps: blast to find homologous sequences, mapping to collect GO-terms associated to blast hits and annotation to assign functional terms to query sequences from the pool of GO terms collected in the mapping step [55]. Function assignment is based on GO database. Sequence data of identified proteins were uploaded as a multiple FASTA file to the Blast2GO software. We performed the blast step against public database NCBI through blastp. Other parameters were kept at default values: e-value threshold of 1e-3 and a recovery of 20 hits per sequence. Furthermore, minimal alignment length (hsp filter) was set to 33 to avoid hits with matching region smaller than 100 nucleotides. QBlast-NCBI was set as Blast mode. Furthermore, we have chosen an annotation configuration with an e-value-Hit-filter of 1.0E-6, Annotation CutOff of 55 and GO weight of 5. For visualizing the functional information (GO categories: Molecular Function and Biological process) we used the analysis tool of the Blast2GO software.

### Protein Domain Analysis of Proteins without Annotation

Six frame translations of the Unigenes were run through the DomainSweep pipeline [56] and the significant and putative hits were collected. For each of the protein/domain databases used, different thresholds and rules were established [56]. Domain hits are listed as ‘significant’

- if two or more hits belong to the same INTERPRO [57] family. The task compares all true positive hits of the different protein family databases grouping together those hits, which are members of the same INTERPRO family/domain.
- if the motif shows the same order as described in PRINTS [58] or BLOCKS [59]. Both databases characterize a protein family with a group of highly conserved motifs/segments in a well-defined order. The task compares the order of the identified true positive hits with the order described in the corresponding PRINTS or BLOCKS entry. Only hits in correct order are accepted.

All other hits above the trusted thresholds are listed as ‘putative’. By comparing the peptides which were identified by mass spectrometry with the six translations, the correct frame and the associated domain information was listed.

## Supporting Information

Table S1 Blast2GO analysis of identified proteins. Spot number, protein annotation, accession number and GO information in all three categories molecular function, biological process and cellular component are listed.
(0.16 MB XLS)
